# Increased global transcription activity as a mechanism of replication stress in cancer

**DOI:** 10.1038/ncomms13087

**Published:** 2016-10-11

**Authors:** Panagiotis Kotsantis, Lara Marques Silva, Sarah Irmscher, Rebecca M. Jones, Lisa Folkes, Natalia Gromak, Eva Petermann

**Affiliations:** 1 Institute of Cancer and Genomic Sciences, University of Birmingham, Edgbaston, Birmingham B15 2TT, UK; 2 Sir William Dunn School of Pathology, University of Oxford, South Parks Road, Oxford OX1 3RE, UK; 3 Department of Oncology, CRUK/MRC Oxford Institute for Radiation Oncology, University of Oxford, Old Road Campus Research Building, Roosevelt Drive, Oxford OX3 7DQ, UK

## Abstract

Cancer is a disease associated with genomic instability that often results from oncogene activation. This in turn leads to hyperproliferation and replication stress. However, the molecular mechanisms that underlie oncogene-induced replication stress are still poorly understood. Oncogenes such as HRAS^V12^ promote proliferation by upregulating general transcription factors to stimulate RNA synthesis. Here we investigate whether this increase in transcription underlies oncogene-induced replication stress. We show that in cells overexpressing HRAS^V12^, elevated expression of the general transcription factor TATA-box binding protein (TBP) leads to increased RNA synthesis, which together with R-loop accumulation results in replication fork slowing and DNA damage. Furthermore, overexpression of TBP alone causes the hallmarks of oncogene-induced replication stress, including replication fork slowing, DNA damage and senescence. Consequently, we reveal that increased transcription can be a mechanism of oncogene-induced DNA damage, providing a molecular link between upregulation of the transcription machinery and genomic instability in cancer.

## Abstract (web summary)

Cancer cells proliferate at high rates and incur replication stress. Here, the authors show that this can be the consequence of oncogene-induced higher transcriptional activity, which, through increased RNA synthesis and R-loop accumulation, results in replication fork slowing and DNA damage.

## Graphical abstract

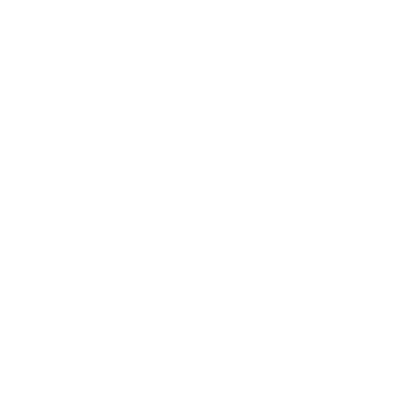

Cancer is a disease of genomic instability, characterized by high mutation rates and genomic rearrangements that ultimately drive aggressiveness and resistance to therapy^1^. One of the mechanisms proposed to cause genomic instability in cancer is replication stress, which occurs when DNA replication fork progression in S phase slows or stalls. This leads to collapse of forks into DNA double-strand breaks (DSBs), as well as incomplete sister chromatid separation in the following mitosis^2^. Markers of spontaneous replication stress are found in tumour samples and cells expressing active oncogenes, and replication stress promotes chromosomal instability, the most common form of genomic instability in sporadic cancers^3^,^4^,^5^,^6^. Spontaneous replication stress is therefore increasingly regarded as a central feature of cancer cells and there is much interest in specifically targeting this phenotype for cancer therapy^7^. However, progress in this field is hindered, because the molecular mechanisms underlying spontaneous replication stress in cells are still largely unknown. This impairs our ability to investigate replication stress *in vitro* and *in vivo*, and to identify potential biomarkers or therapeutic targets.

How can mechanisms of spontaneous replication stress be identified? The overexpression of oncogenes such as RAS, MOS, MYC, CDC25A or CYCLIN E is sufficient to induce replication stress in cultured cells^4^,^5^,^8^,^9^,^10^,^11^. These oncogenes all act in the growth factor signalling pathways that stimulate proliferation by promoting cell growth and division. Molecular changes associated with increased proliferation are therefore prime candidates for causing replication stress. Indeed, we and others have reported that CYCLIN E-induced replication stress results from accelerated S-phase entry and increased replication initiation during S phase^9^,^12^. So far, however, little attention has been paid to the fact that oncogenes such as RAS and MYC do not only activate the cell cycle machinery but also promote cell growth through the activation of transcription and protein translation^13^,^14^,^15^,^16^. Overexpressed c-MYC acts as a ‘universal amplifier', stimulating transcription by all three RNA polymerases^14^,^17^,^18^. Oncogenic RAS promotes transcription through the mitogen-activated protein kinase extracellular signal-regulated kinase (ERK), which activates transcription factors such as TIFIA (RRN3), UBTF, TIFIIIB (BRF1) and TATA-box binding protein (TBP)^14^,^15^,^19^, and can also promote transcription through other factors such as TERT^20^. Moreover, components of the general transcription machinery itself are found mutated, differentially expressed and deregulated across a variety of cancers^14^,^21^.

Upregulation of transcription in cancer cells has the potential to be a direct cause of replication stress as interference between transcription and replication leads to replication fork slowing and genomic instability^22^,^23^. This can result from direct collisions or torsional stress between the two active protein complexes^24^. Another important source of replication stress is the collision of the replication machinery with RNA–DNA hybrids (R-loops) where the nascent RNA has re-annealed with the template^25^. Deregulated transcription is a common feature of cancers, but its importance for replication stress and consequent genomic instability has not been examined.

Here we use HRAS^V12^ overexpression^5^,^26^ to investigate whether the oncogene-induced increase in transcription is a mechanism of endogenous replication stress. We report that increased transcription activity in cells expressing HRAS^V12^ causes replication stress, as high levels of RNA synthesis and transcription intermediates interfere with replication fork progression. Replication stress in these cells depends on R-loop accumulation and on increased expression of the general transcription factor TBP. Importantly, overexpression of TBP alone induces replication stress and genomic instability. Our data suggest that increased transcription activity is a mechanism contributing to replication stress in cancer.

## Results

### HRAS^V12^ increases nascent RNA synthesis and R-loop formation

**Figure 1:**
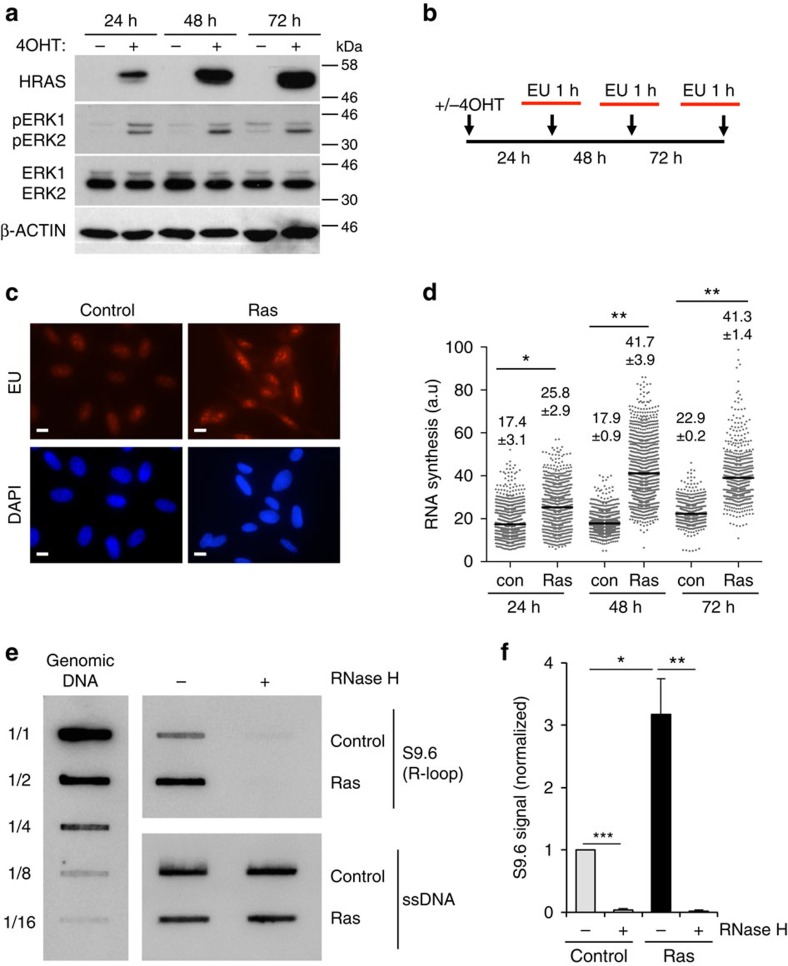
HRAS^V12^ overexpression increases transcription activity. (**a**) Protein levels of HRAS, pERK1/2, ERK1/2 and β-ACTIN in BJ-HRASV12 cells after RAS induction for the times indicated. (**b**) EU incorporation (1 h) was used to measure nascent RNA synthesis after RAS induction for the times indicated. (**c**) Representative images of EU staining (red) after RAS induction for 72 h. (**d**) Quantification of nuclear EU intensity after RAS induction for the times indicated. *N*=4 (48 and 72 h), *N*=5 (24 h). (**e**) RNA/DNA hybrid slot blot of genomic DNA from BJ-HRAS^V12^ cells after RAS induction for 72 h, ±RNase H. S9.6 antibody was used to detect RNA/DNA hybrids (top panel) with single-strand DNA antibody (bottom panel) as a loading control. Serial dilutions of genomic DNA (1/1=4 μg) were probed with S9.6 antibody for standards (left panel). (**f**) Fold enrichment in RNA/DNA hybrids compared with control. *N*=3. Means ±s.e.m. (bars) are shown. Student's *t*-test, *P<0.05, **P<0.01 and ***P<0.001. Scale bars, 10 μm.

To investigate whether increased transcription activity contributes to oncogene-induced replication stress, we used immortalized human fibroblasts that have been stably transfected with pBabe-HRAS^V12^-ER^TAM^, to express tamoxifen-inducible HRAS^V12^ (BJ-hTert HRASV12^ER-TAM^)^27^. HRAS^V12^-ER^TAM^ has been well characterized as a system for inducing oncogenic RAS^26^,^28^,^29^. Addition of 4-hydroxytamoxifen (4OHT) led to HRAS^V12^ accumulation, which activated mitogen-activated protein kinase signalling as evidenced by ERK1/2 phosphorylation (Fig. 1a). Cells proliferated slightly faster for up to 6 days followed by growth arrest, which was previously shown to result from DNA damage-induced apoptosis and senescence^4^,^5^ (Supplementary Fig. 1a–e). When using immortalized BJ fibroblasts that were not expressing 4OHT-inducible HRAS^V12^, 4OHT treatment did not affect any of the phenotypes investigated in this study (Supplementary Fig. 1f–i).

First, we investigated the effect of oncogenic HRAS on global transcription activity. For this, we quantified nascent RNA synthesis using nuclear incorporation of the modified RNA precursor 5-ethynyluridine (EU) for 1 h (Fig. 1b,c). RNA synthesis increased rapidly after HRAS^V12^ induction and was elevated more than twofold after 48 and 72 h HRAS^V12^ induction (Fig. 1d). RNA synthesis after HRAS^V12^ induction was higher than the highest activity observed in control cells (Fig. 1d), arguing against changes in cell cycle distribution as the sole explanation for the increase. Seventy-two hours of HRAS^V12^ induction was used for most subsequent experiments.

**Figure 2:**
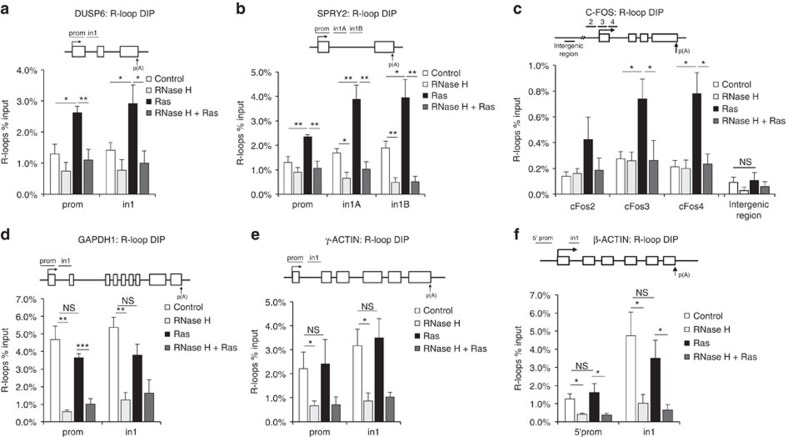
HRAS^V12^ overexpression increases R-loop formation. DIP analysis of R-loop induction on the *DUSP6* (**a**), *SPRY2* (**b**), *C-FOS* (**c**), *GAPDH* (**d**), *γ-ACTIN* (**e**) and *β-ACTIN* (**f**) genes in BJ-HRAS^V12^ cells after RAS induction for 72 h. Intergenic region upstream of *C-FOS* gene (**c**) served as a background control. Values are percentage of input. *N*=5. Primer positions are shown in upper panels of **a**–**f** (white boxes represent exons). Means ±s.e.m. (bars) are shown. Student's *t*-test, *P<0.05, **P<0.01 and ***P<0.001. NS, nonsignificant.

We next used the S9.6 antibody that detects RNA/DNA hybrids^30^,^31^, to test whether R-loop formation was increased in HRAS^V12^-overexpressing cells. First, we performed slot blot analysis of isolated genomic DNA^32^, which revealed a threefold increase in R-loops after 72 h HRAS^V12^ induction (Fig. 1e,f). The S9.6 signal could be removed by treatment with recombinant RNase H, supporting that it was specific to R-loops. We then used S9.6 immunoprecipitation of RNA/DNA hybrids from cells (DNA immunoprecipitation (DIP))^33^ after 72 h HRAS^V12^ induction, to investigate the distribution of R-loops across RAS target versus control genes (Fig. 2a–f). We used quantitative PCR (qPCR) to quantify R-loop distribution on *DUSP6*, *SPRY2* and *C-FOS*, genes that are upregulated by activated RAS (Fig. 2a–c and Supplementary Fig. 2a). We observed an increased R-loop formation on the promoter-proximal and selected intron regions of all three genes. DIP analysis of *C-FOS* showed that in line with previously described R-loop accumulation in actively transcribed genes^33^, R-loops were significantly increased over the transcribed regions of the gene (Fig. 2c and also see Supplementary Table 1 for PCR primer sequences). We also quantified R-loop formation on non-RAS target control genes *GAPDH1*, *ACTB* (β-ACTIN) and *ACTG* (γ-ACTIN). We observed no increase in R-loops across any of these genes (Fig. 2d–f). RNase H treatment confirmed that DIP specifically detected R-loops (Fig. 2a–f). RNase A treatment confirmed that DIP signal was not due to annealing of free RNA species to DNA during sample preparation or to S9.6 antibody recognizing double-stranded RNA (Supplementary Fig. 2b–e). These data support that stimulation of transcription by HRAS^V12^ results in increased R-loop formation.

### HRAS^V12^ causes replication stress and G1 53BP1 foci

**Figure 3:**
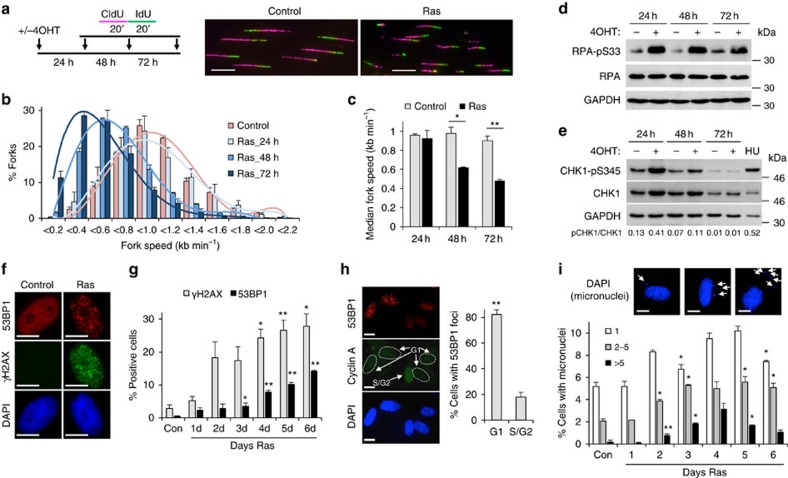
HRAS^V12^ causes replication stress and genomic instability. (**a**) Top: DNA fibre labelling was performed in BJ-HRAS^V12^ cells after RAS induction for 24, 48 and 72 h. Bottom: representative images of DNA fibres after 72 h RAS induction. (**b**) Distributions of replication fork speeds after RAS induction. *N*=3. (**c**) Median replication fork speeds after RAS induction. *N*=3. (**d**) Protein levels of phospho-S33 RPA32, RPA32 and GAPDH after RAS induction. (**e**) Protein levels of phospho-S345 CHK1, CHK1 and GAPDH after RAS induction. Hydroxyurea (HU, 2 mM for 24 h) was used as a positive control. (**f**) Representative images of γH2AX and 53BP1 foci after RAS induction for 96 h. (**g**) Percentages of cells containing more than 8 γH2AX or 53BP1 foci after RAS induction for 96 h. *N*=2. Asterisks compare with control. (**h**) Cell cycle distribution of 53BP1-positive cells after 96 h RAS induction as determined by co-staining with Cyclin A. Right panel: representative images; left panel: quantification. *N*=3. (**i**) Representative images of cells with micronuclei and percentages of cells containing micronuclei after RAS induction for 1–6 days. *N*=2. Asterisks compare with control. Means ±s.e.m. (bars) are shown. Student's *t*-test, *P<0.05 and **P<0.01. Scale bars, 10 μm.

HRAS^V12^ overexpression caused replication fork slowing after 48 h, indicative of replication stress (Fig. 3a–c) and induced phosphorylation of replication stress response factors RPA32 (serine 33) and CHK1 (serine 345) (Fig. 3d,e). These modifications were already evident at 24 h HRAS^V12^ induction, which may also reflect the proposed role for RPA and ATR in the transcription stress response^34^. As expected, fork slowing was associated with nuclear foci formation of DNA damage markers γH2AX and 53BP1 (Fig. 3f,g). One consequence of replication fork slowing is mitotic entry with under-replicated DNA, which leads to micronuclei formation and appearance of 53BP1 bodies in the following G1 phase^35^,^36^. Accordingly, 53BP1 foci increased after 4 days HRAS^V12^ induction and were mostly found in G1 cells (Fig. 3h). Similarly, micronuclei formation peaked at 4 days HRAS^V12^ induction (Fig. 3i). This suggests that HRAS^V12^-induced DNA damage requires mitotic progression, as was previously reported for CYCLIN E^10^. Four days of HRAS^V12^ induction was used for subsequent investigation of 53BP1 foci formation.

### Transcription promotes HRAS^V12^-induced replication stress

**Figure 4:**
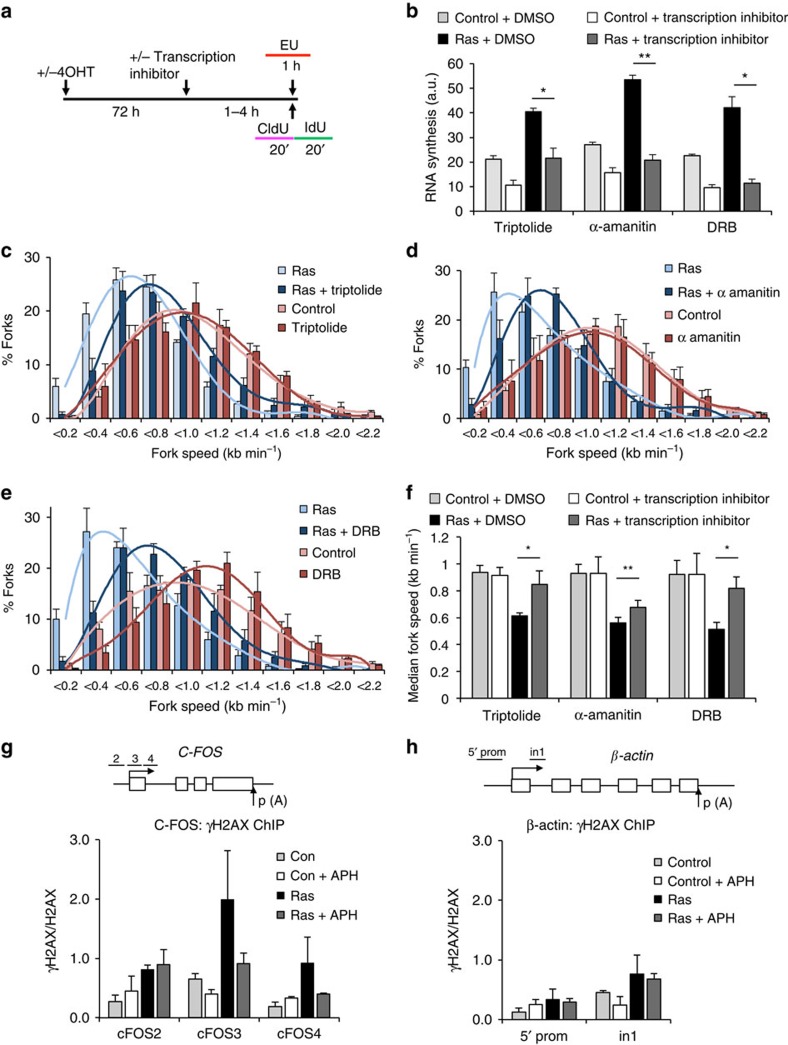
HRAS^V12^-induced replication stress is promoted by ongoing transcription. (**a**) Seventy-two hours after RAS induction, BJ-HRAS^V12^ cells were incubated with transcription inhibitors triptolide, α-amanitin, DRB or dimethylsulfoxide (DMSO) for 100 min to 4 h before and during EU or DNA fibre labelling. (**b**) Quantification of nascent RNA synthesis by EU incorporation in cells treated with transcription inhibitors 72 h after RAS induction. *N*=2 (con + triptolide, con + DRB), *N*=3 (all other samples). (**c**–**e**) Distributions of replication fork speeds in cells treated with transcription inhibitors 72 h after RAS induction. *N*=3 (DRB, control + α-amanitin, control + triptolide), *N*=5 (other samples). (**f**) Median replication fork speeds in cells treated with transcription inhibitors 72 h after RAS induction. (**g**) ChIP analysis of γH2AX versus total H2AX levels on the promoter of the *C-FOS* gene 72 h after RAS induction. + APH samples were treated with 0.5 μM Aphidicolin for 2 h before ChIP. *N*=3. (**h**) ChIP analysis of γH2AX versus total H2AX levels on the *β-ACTIN* gene 72 h after RAS induction as in g. *N*=3. qPCR primer positions are shown in upper panels. Means ±s.e.m. (bars) are shown. Student's *t*-test, *P<0.05 and **P<0.01.

To test whether increased transcription causes replication stress in cells harbouring HRAS^V12^, we transiently inhibited RNA synthesis using small molecule inhibitors before measuring replication fork progression. To minimize effects on gene expression, incubations were kept short at 100 min for triptolide and 5,6-dichloro-1-β-D-ribofuranosyl-1H-benzimidazole (DRB) and 4 h for α-amanitin (Fig. 4a). These treatments inhibited ongoing RNA synthesis (Fig. 4b) but did not affect protein levels of HRAS^V12^ or levels of proteins with short half lives such as CYCLIN B1 and p53 (Supplementary Fig. 3a,b). All three transcription inhibitors increased replication fork speeds specifically in the presence of HRAS^V12^ (Fig. 4c–f). Although such short incubations with transcription inhibitors may not be sufficient to reverse all effects of transcription, these data suggest that RAS-induced replication stress is promoted by active RNA synthesis. DRB and triptolide rescued replication more effectively than α-amanitin, suggesting that effective inhibition of early stages of transcription removes more obstacles to replication forks than does inhibiting transcription during elongation (Fig. 4f).

We previously observed that increased CDK activity and new origin firing underlies replication stress in cells overexpressing CYCLIN E^12^. In contrast, inhibiting new origin firing using CDK inhibitor roscovitine could not rescue HRAS^V12^-induced replication fork slowing (Supplementary Fig. 3c–e). Together, our data suggest that replication stress in cells expressing HRAS^V12^ is promoted by transcription but not by CDK activity.

To test whether HRAS^V12^-induced DNA damage was transcription dependent, we first incubated cells with DRB for 100 min and stained for 53BP1 24 h later. DRB reduced 53BP1 foci formation; however, its impact was limited by the short incubation time (Supplementary Fig. 3f,g). We therefore used γH2AX chromatin immunoprecipitation (ChIP) to test whether transcription and R-loop formation was associated with DNA damage in cells harbouring HRAS^V12^. Indeed, we observed an increase in γH2AX signal over the transcribed promoter-proximal region of the *C-FOS* gene, correlating with strong induction of R-loops, 72 h after HRAS^V12^ induction (Figs 2c and 4g). This γH2AX induction was replication dependent, as it could be prevented by blocking replication with Aphidicolin (Fig. 4g and Supplementary Fig. 1d). In contrast, we did not detect an increase in replication-dependent γH2AX levels across the intron 1 region of the *β-ACTIN* gene (Fig. 4h). This suggests that HRAS^V12^ triggers R-loop-associated DNA damage that also depends on replication.

### R-loops promote HRAS^V12^-induced replication stress

**Figure 5:**
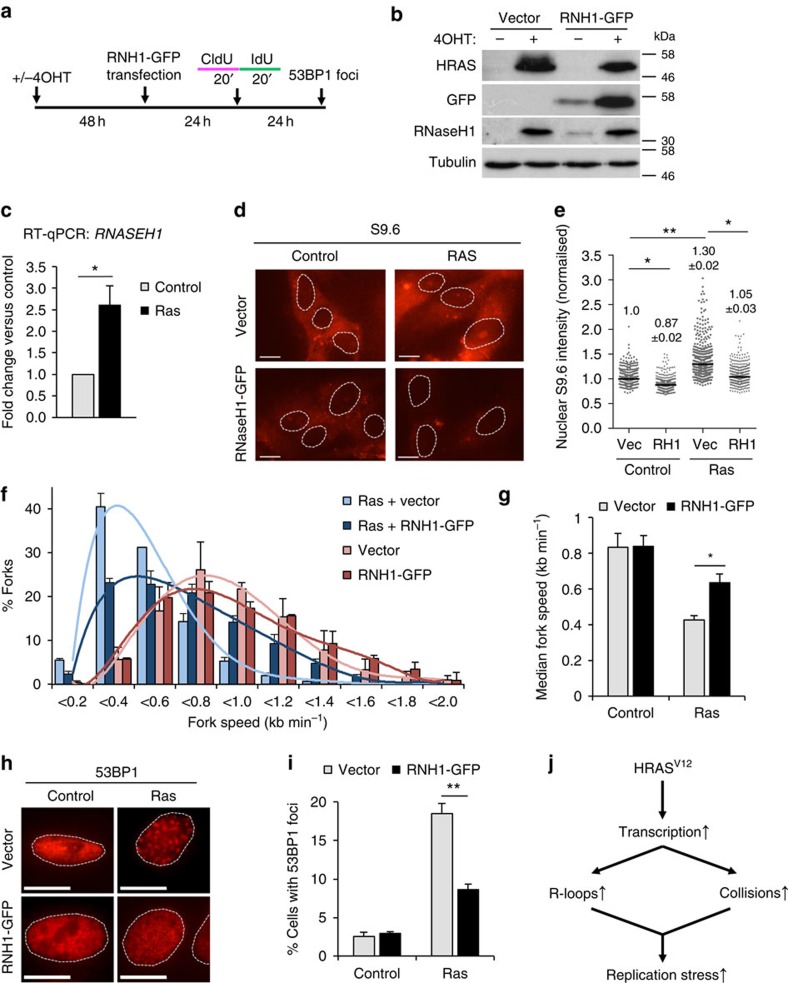
HRAS^V12^ induces replication stress through R-loop formation. (**a**) After RAS induction for 48 h, BJ-HRAS^V12^ cells were transfected with pCMV6-AC-RNaseH1-GFP or empty vector and processed for DNA fibre analysis or 53BP1 foci staining after 24 or 48 h. (**b**) Protein levels of HRAS, GFP, RNaseH1 and TUBULIN (loading control) 72 h after RAS induction and 24 h after transfection with pCMV6-AC-RNaseH1-GFP. (**c**) *RNASEH1* mRNA quantification by quantitative reverse transcriptase–PCR after 72 h RAS induction. *RNASEH1* mRNA levels were normalized to *GAPDH* and control. (**d**) Representative images of S9.6 immunostaining ±pCMV6-AC-RNaseH1-GFP 72 h after RAS induction. (**e**) Quantification of nuclear S9.6 intensity ±pCMV6-AC-RNaseH1-GFP 72 h after RAS induction. *N*=3. (**f**) Distribution of fork speeds ±pCMV6-AC-RNaseH1-GFP 72 h after RAS induction. *N*=3. (**g**) Median replication fork speeds ±pCMV6-AC-RNaseH1-GFP 72 h after RAS induction. *N*=3. (**h**) Representative images of cells with 53BP1 foci, ±pCMV6-AC-RNaseH1-GFP 96 h after RAS induction. (**i**) Percentage of cells displaying more than 8 53BP1 foci, ±pCMV6-AC-RNaseH1-GFP 96 h after RAS induction. *N*=3. (**j**) Model of how HRAS^V12^ causes replication stress via increasing transcription. Means ±s.e.m. (bars) are shown. Student's *t*-test, *P<0.05 and **P<0.01. Scale bars, 10 μm.

We next decided to further investigate the role of R-loops in HRAS^V12^-induced replication stress. We used transient transfection to express green fluorescent protein (GFP)-tagged human RNaseH1, an enzyme that degrades RNA/DNA hybrids on overexpression^37^ (Fig. 5a,b). Interestingly, we observed that protein and messenger RNA levels of endogenous RNaseH1 were elevated in cells overexpressing HRAS^V12^, suggesting an increased requirement for R-loop processing activities (Fig. 5b,c). The specificity of RNaseH1 antibody was verified using small interfering RNA (siRNA) depletion of RNaseH1 (Supplementary Fig. 4a). Overexpression of GFP-RNaseH1 reduced R-loop levels in the nucleus, as indicated by S9.6 immunostaining (Fig. 5d,e and also see Supplementary Fig. 5 for validation of immunostaining method). As the expression construct contains the RNaseH1 mitochondrial targeting sequence, mitochondrial R-loops were also reduced (Fig. 5d). RNaseH1 overexpression effectively improved replication fork progression in cells harbouring HRAS^V12^ (Fig. 5f,g). GFP-RNaseH1 also decreased HRAS^V12^ induction of 53BP1 foci (Fig. 5h,i) and DSB induction measured by pulse-field gel electrophoresis (Supplementary Fig. 4b,c). GFP-RNaseH1 overexpression did not affect hydroxyurea-induced 53BP1 foci formation or the cell cycle profile (Supplementary Fig. 4d,e). These data support that HRAS^V12^-induced replication stress is promoted by the presence of R-loops.

We also tested whether some of the replication stress caused by HRAS^V12^ might be due to ribonucleotide (NTP) depletion as a result of increased RNA synthesis. Supplementing growth medium with ribonucleosides improved fork speeds and reduced 53BP1 foci formation in the presence of HRAS^V12^ (Supplementary Fig. 6a–c). However, HPLC quantification showed no reduction in NTP levels or dNTP levels as detectable (Supplementary Fig. 6d,e). This is in line with previous reports demonstrating that nucleoside supplementation can rescue fork progression even in the absence of detectable nucleotide depletion^38^. Our data thus suggest that transcription contributes to HRAS^V12^-induced replication stress predominantly via direct conflicts with transcription machinery and R-loops (Fig. 5j).

### HRAS^V12^-induced replication stress depends on TBP

**Figure 6:**
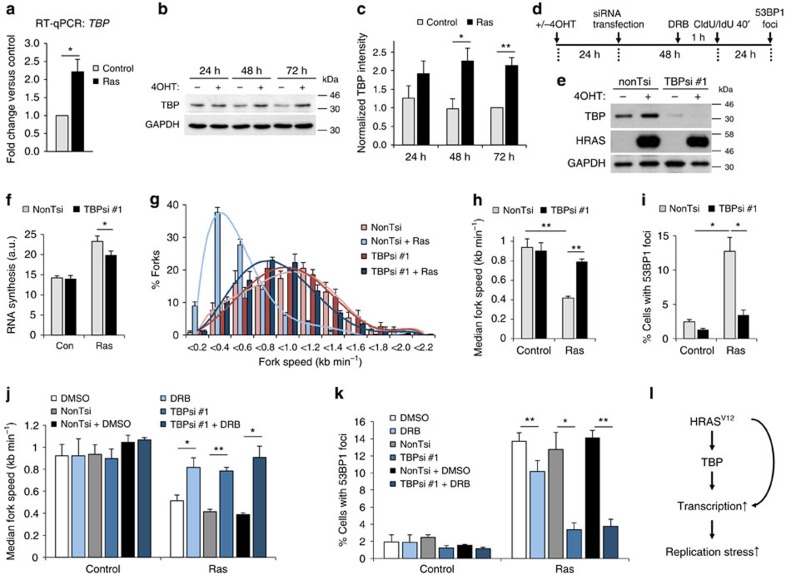
TBP is an effector in HRAS^V12^-induced replication stress. (**a**) *TBP* mRNA quantification by quantitative reverse transcriptase–PCR in BJ-HRAS^V12^ cells 72 h after RAS induction. *TBP* mRNA levels were normalized to *GAPDH* and control. (**b**) Protein levels of TBP and β-ACTIN after RAS induction for the times indicated. (**c**) Densitometry quantification of TBP levels based on western blotting as in **b** after RAS induction for the times indicated. Values were normalized to 72 h control. *N*=3 (24 and 48 h), *N*=6 (72 h). (**d**) Twenty-four hours after RAS induction, cells were transfected with TBP siRNA (TBPsi #1) or control siRNA (nonTsi). Cells were processed for DNA fibre analysis or western blotting 48 h later and for 53BP1 staining 24 h later. (**e**) Protein levels of TBP, HRAS and GAPDH (loading control) 72 h after RAS induction and 48 h after siRNA transfection. (**f**) Quantification of nascent RNA synthesis by EU incorporation ±TBPsi #1 72 h after RAS induction. *N*=3. (**g**) Distribution of replication fork speeds ±TBPsi #1 72 h after RAS induction. *N*=3. (**h**) Median replication fork speeds ±TBPsi #1 72 h after RAS induction. *N*=3. (**i**) Percentages of cells containing more than eight 53BP1 foci, ±TBPsi #1 96 h after RAS induction. *N*=3. (**j**) Median replication fork speeds in cells treated with TBPsi #1 and DRB 72 h after RAS induction, compared with TBPsi #1 or DRB alone. *N*=3. (**k**) Percentages of cells treated with TBPsi #1 and DRB containing more than eight 53BP1 foci after 96 h after RAS induction, compared with TBPsi #1 or DRB alone. *N*=3. (**l**) Model for the role of TBP in HRAS^V12^-induced replication stress. Means ±s.e.m. (bars) are shown. Student's *t*-test, *P<0.05, **P<0.01 and ***P<0.001.

We next turned our attention to transcription factors that may promote RNA synthesis and therefore replication stress downstream of HRAS^V12^. TBP expression is induced by RAS signalling in a number of human, murine and *Drosophila* cell types^19^,^39^,^40^, and TBP mRNA and protein levels were accordingly increased after HRAS^V12^ induction (Fig. 6a–c). We used siRNA depletion to test whether TBP promotes HRAS^V12^-induced replication stress (Fig. 6d,e). TBP depletion decreased nascent RNA synthesis in HRAS^V12^-expressing cells (Fig. 6f). Importantly, TBP depletion also prevented HRAS^V12^-induced fork slowing (Fig. 6g,h) and TBP-depleted cells displayed fewer HRAS^V12^-induced 53BP1 foci (Fig. 6i). Similar results were obtained using a different siRNA sequence targeting TBP (Supplementary Fig. 7a–e). We noticed that the alternative TBP siRNA also reduced RNA synthesis and replication fork speeds in control cells, suggesting that generally low RNA synthesis may affect replication (Supplementary Fig. 7c,d). TBP depletion did not prevent hydroxyurea-induced 53BP1 foci (Supplementary Fig. 6f) and did not affect the cell cycle profile of 53BP1 foci-positive cells, suggesting that the reduction in foci was not due to a G2 arrest (Supplementary Fig. 7g).

To test whether TBP acts in the same pathway as ongoing RNA synthesis, we combined TBP siRNA with DRB treatment. Combining both treatments rescued replication fork progression and 53BP1 foci formation to a similar extent as TBP depletion alone (Fig. 6j,k and Supplementary Fig. 8a). To further test whether ribonucleoside addition was affecting replication stress via a different pathway to transcription^41^, we combined TBP siRNA and DRB with nucleoside supplementation. Compared with transcription inhibition alone, exogenous nucleosides had no additional effect on either the rescue of replication fork progression or the reduction in 53BP1 foci (Supplementary Fig. 8b–e).

These data suggest that the main pathway of HRAS^V12^-induced replication stress is via upregulation of transcription, and that TBP is involved in this upregulation of transcription and the replication fork slowing and genomic instability that result from it (Fig. 6l).

### TBP overexpression alone causes replication stress

**Figure 7:**
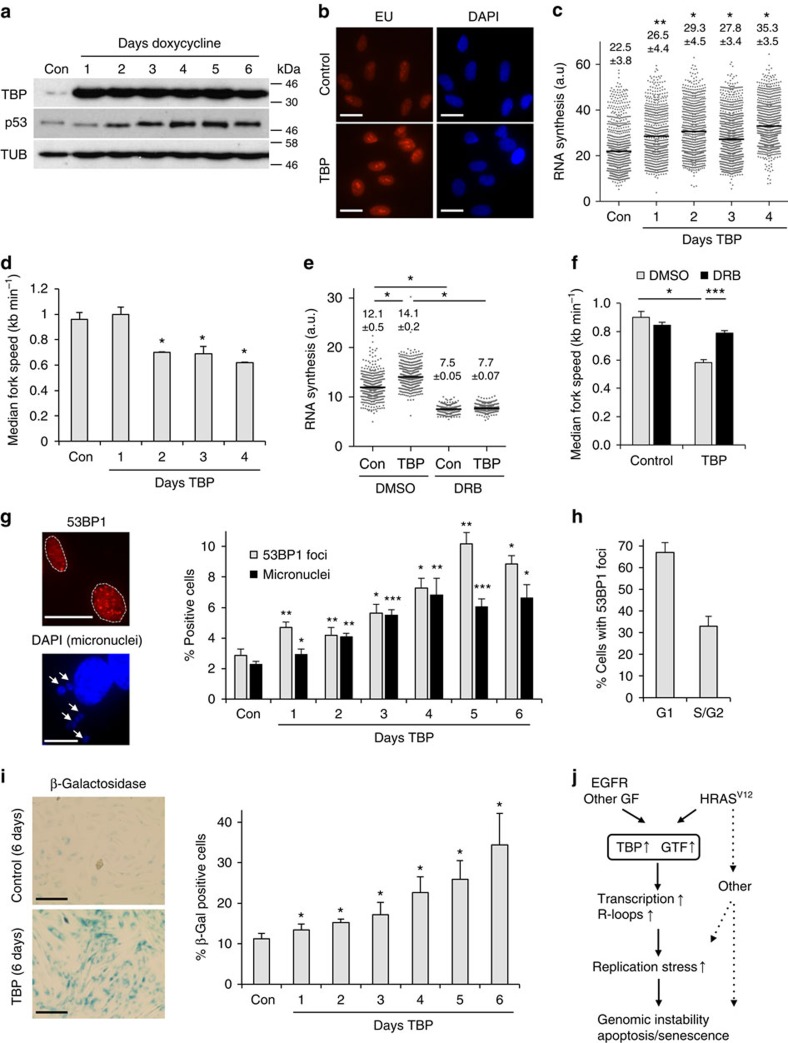
TBP overexpression causes replication stress and senescence. (**a**) Protein levels of TBP, p53 and TUBULIN (loading control) in BJ-TBPind cells treated with doxycycline for 1–6 days, to induce TBP overexpression. No doxycycline was used as a control. (**b**) Nascent RNA synthesis as measured by EU incorporation after TBP induction for 3 days. Scale bars, 10 μm. (**c**). Quantification of nuclear EU intensity after TBP induction for 1–4 days. *N*=4 (con, day 1, 2 and 4), *N*=5 (day 3). (**d**) Median replication fork speeds in after TBP induction. *N*=2 (day 1, 2 and 4), *N*=4 (con, day 3). Asterisks compare with control. (**e**) Nascent RNA synthesis as measured by EU incorporation in cells treated with DRB or dimethylsulfoxide (DMSO) (control) for 100 min, 72 h after TBP induction. *N*=3. (**f**) Median replication fork speeds in BJ-TBPind cells treated with DRB 72 h after TBP induction. *N*=3. (**g**). Percentage of cells displaying more than eight 53BP1 foci or micronuclei after TBP induction. Right panel: representative images of cells with 53BP1 foci and micronuclei. Asterisks compare with control. *N*=2–7. (**h**) Cell cycle distribution of 53BP1-positive cells 96 h after TBP induction as determined by co-staining with Cyclin A. *N*=2. (**i**) Representative images and percentages of β-galactosidase staining after TBP induction for 1–6 days. Scale bars, 100 μm. (**j**) Model of how HRASV^12^ and other growth factor oncogenes such as epidermal growth factor receptor (EGFR) induce replication stress by increasing transcription through TBP and other transcription factors. Additional mechanisms, such as reactive oxygen species, may also contribute to HRASV^12^-induced DNA damage. Means ±s.e.m. (bars) are shown. Student's *t*-test, *P<0.05, **P<0.01 and ***P<0.001.

We reasoned that if TBP was a downstream effector in HRAS^V12^-induced replication stress, then overexpression of TBP alone should cause replication stress. We therefore generated BJ-hTert fibroblasts for doxycycline-inducible overexpression of TBP (BJ-TBPind; Fig. 7a). Inducing TBP overexpression over several days led to a steady increase in RNA synthesis activity as measured by EU incorporation (Fig. 7b,c). Importantly, the increase in transcription activity during TBP overexpression was accompanied by replication fork slowing, consistent with replication stress (Fig. 7d and Supplementary Fig. 9a). Similar results were observed in human MRC5 fibroblasts overexpressing TBP (Supplementary Fig. 9b–d). To test whether TBP-induced fork slowing depended on ongoing transcription, we treated TBP-overexpressing cells with DRB for 100 min, to inhibit nascent RNA synthesis (Fig. 7e). DRB treatment rescued the TBP-induced fork slowing (Fig. 7f and Supplementary Fig. 9e), supporting that TBP overexpression causes replication stress through nascent RNA synthesis.

We next characterized the effect of TBP overexpression on DNA damage, genomic instability and proliferation. Similar to HRAS^V12^-expressing cells, cells overexpressing TBP displayed increased formation of micronuclei and 53BP1 foci (Fig. 7g), and 53BP1 foci were mostly found in G1 cells (Fig. 7h). Finally, TBP overexpression eventually led to growth arrest and senescence, similar to that caused by HRAS^V12^ (Fig. 7i and Supplementary Fig. 9f,g). In agreement with this, p53 levels were increased after TBP induction (Fig. 7a and also see Supplementary Fig. 10 for original images of western blottings).

Thus, our data show that TBP alone is able to increase transcription activity and cause replication stress with features that resemble oncogene-induced replication stress.

### TBP expression and replication stress in cancer

Finally, we investigated the relationship of *TBP* and *RNASEH1* mRNA expression with oncogenes and replication stress markers in tumour samples using The Cancer Genome Atlas (TCGA) data sets^42^,^43^,^44^,^45^,^46^,^47^,^48^,^49^,^50^ (Table 1). In a number of cancers, *TBP* or *RNASEH1* expression correlated positively with expression of *RAS* or *MYC* oncogenes and the *CHEK1* or *CHEK2* checkpoint kinases, which are activated by oncogene-induced replication stress^4^,^5^. These data support that the relationship between oncogenes, TBP, R-loops and replication stress could be present in cancer tissues.

## Discussion

We report that increased transcription is a new mechanism of oncogene-induced replication stress. Overexpression of oncogenic HRAS^V12^ increases RNA synthesis and R-loop formation, and this directly contributes to replication stress induced by HRAS^V12^. HRAS^V12^-induced replication stress is mediated by the general transcription factor TBP, which is a downstream target of RAS signalling. Accordingly, overexpression of TBP itself causes replication stress and genomic instability. The transcription machinery is frequently deregulated in cancer cells^21^ and our data suggest that transcription-associated replication stress could be an important mechanism promoting genomic instability in cancer (Fig. 7j).

Markers of replication stress are observed in early tumours and cancers and other conditions of high proliferation, such as stem cell reprogramming or viral infection^51^,^52^. Recent studies have provided first candidates for the mechanisms causing such endogenous replication stress. So far, all of these have involved deregulation of the cell cycle, including re-replication^53^ and premature or increased replication initiation, resulting in depletion of nucleotides or replication enzymes^9^,^10^,^12^. In addition, reactive oxygen species play important roles in DNA damage in cancer, but whether they can cause replication stress is still uncertain^54^,^55^. Our data support that increased transcription is another pathway causing endogenous replication stress, which may act in parallel or independently of cell cycle deregulation. HRAS^V12^ overexpression did increase the density of active replication origins, which could be reversed by CDK inhibition. However, CDK inhibition was unable to relieve HRAS^V12^-induced replication fork slowing, suggesting that increased replication initiation is not a cause but a consequence of HRAS^V12^-induced replication stress (Supplementary Fig. 3d,e). Our data suggest that HRAS^V12^ causes replication stress by a mechanism that is different from oncogenes such as CYCLIN E and CDC25A^10^,^12^.

Our data support that HRAS^V12^ overexpression promotes accumulation of R-loops, which can be a major cause for replication fork slowing and DNA breakage^23^. An elegant study recently reported that loss of tumour suppressors BRCA1 or BRCA2 increases R-loop levels, because these proteins act to prevent R-loop formation^56^. Our findings add an important new angle to this observation, showing that oncogenes can induce R-loops, which suggests that increased R-loop levels might also be common in cancer cells that are proficient in BRCA1 or BRCA2. Interestingly, we observed increased protein levels of endogenous RNaseH1 in cells overexpressing HRAS^V12^ (Fig. 5b,c). RNaseH1 and RNaseH2 are the main RNase activities counteracting R-loop formation and upregulation of these enzymes may be a response of cancer cells to increased R-loop levels^23^. Although the biology of increased RNaseH1 expression in response to HRAS^V12^ requires further investigation, it supports the idea that oncogenic replication stress is strongly connected with R-loop metabolism.

One exciting implication of our findings is the potential to discover new factors involved in promoting replication stress. Components of the transcription machineries are found overexpressed in cancer^21^,^57^. TBP is one of several general transcription factors that have been implicated in oncogene-induced cell transformation^39^,^58^. The *TBP* promoter contains binding sites for oncogenic transcription factors and TBP is differentially expressed in cancers (Table 1)^59^. RAS has been reported to upregulate TBP expression through RAF-MEK and RALGDS signalling^19^, and TBP levels are also increased by other growth factor signalling pathways such as epidermal growth factor/epidermal growth factor receptor^59^,^60^ (Fig. 7j). We report here that increased TBP levels cause replication stress and markers of genomic instability. TBP expression also correlates with the expression of MYC, RAS and checkpoint kinases in a number of cancers (Table 1). As TBP overexpression did not increase RNA synthesis as strongly as HRAS^V12^ overexpression and TBP depletion did not completely rescue HRAS^V12^-induced replication stress, other transcription factors such as UBTF may also be involved downstream of HRAS^V12^ (Fig. 7j).

A previous report showed that TBP is important for HRAS^V12^ transforming function, and that TBP overexpression alone promotes anchorage-independent growth and tumour growth *in vivo*^49^. Our data presented here support the idea that TBP promotes the oncogenic phenotype by increasing transcription activity and replication stress. There is however no evidence that TBP itself is an oncogene, as TBP overexpression promoted tumour growth but not tumourigenesis itself^49^. Some cancer types show recurrent mutations in TBP that affect the length of the amino-terminal glutamine-rich motif (for example, Q72dup in diffuse large B-cell lymphoma and adrenocortical carcinoma)^61^. It is yet unknown whether such mutations have pathogenic relevance in cancer and whether they could be oncogenic.

Finally, increased RNA synthesis and elevated levels of R-loops could be correlated with replication stress in cancer. Identifying the molecular mechanisms that directly cause spontaneous replication stress should therefore help to predict and detect replication stress more accurately in cells and tissues. Nascent RNA synthesis can only be measured in live cells but increased expression of transcription factors and RNA polymerase subunits^21^, as well as increased R-loops and expression of RNases H1 or H2, might be promising candidates for markers of transcription-associated replication stress.

## Methods

### Cell lines and reagents

Human BJ-hTert HRASV12^ER-TAM^ (Agami and de Vita labs^26^,^27^) BJ-hTert and MRC5 fibroblasts (ATCC) were authenticated using 8-locus short tandem repeat (STR) profiling (LGC Standards). Cells were confirmed to be free of Mycoplasma infection and were grown in DMEM medium (Sigma) with 10% fetal bovine serum (Sigma) supplemented with L-glutamine (Gibco) in a humidified atmosphere containing 5% CO_2_. For BJ-TBPind cells, human TBP complementary DNA (Origene, SC118124) was inserted into a pInducer20 lentivirus construct to generate TBP-pInducer20 and infected BJ-hTert cells were selected with 500 μg ml^−1^ G418 (Gibco). HRAS^V12^ was induced with 333 nM 4OHT (Sigma) and TBP expression was induced with 2 μg ml^−1^ doxycycline (Sigma). 4OHT or doxycycline remained present during all experiments.

DRB (100 μM) was from Sigma, and triptolide (1 μM) and α-amanitin (10 μg ml^−1^) were from Tocris Bioscience. Ribonucleosides (adenosine, guanosine, uridine and cytidine, 10 μM) were obtained from Sigma. Roscovitine (25 μM) was from Sigma. Hydroxyurea (2 mM) was from Acros Organics, bleomycin (10 μg ml^−1^) was from Sigma and cycloheximide (100 μg ml^−1^) was from Calbiochem.

### DNA fibre analysis

Cells were pulse labelled with 25 μM CldU and 250 μM IdU for 20 min and harvested. DNA fibre spreads were prepared by spotting 2 μl of cells (5 × 10^5^ cells per ml in PBS) onto microscope slides followed by lysis with 7 μl of 0.5% SDS, 200 mM Tris-HCl pH 7.4 and 50 mM EDTA. Slides were tilted and DNA spreads fixed in methanol/acetic acid (3:1). HCl-treated fibre spreads were incubated with rat anti-bromodeoxyuridine (detects CldU, BU1/75, AbD Serotec, 1:1,000) and mouse anti-bromodeoxyuridine (detects IdU, B44, Becton Dickinson, 1:500) for 1 h, fixed with 4% paraformaldehyde (PFA) to increase staining intensity and incubated with anti-rat IgG AlexaFluor 555 and anti-mouse IgG AlexaFluor 488 (Molecular Probes) for 1.5 h. Images were acquired on an Nikon E600 microscope using a Nikon Plan Apo × 60 (1.3 numerical aperture) oil lens, a Hamamatsu digital camera (C4742-95) and the Volocity acquisition software (Perkin Elmer). Images were analysed using ImageJ (http://rsbweb.nih.gov/ij/). In each independent experiment, at least 300 fibres were measured per condition.

### Immunofluorescence

Cells were washed and fixed as follows. γH2AX and 53BP1: CSK buffer (10 mM PIPES, 300 mM sucrose, 100 mM NaCl and 3 mM MgCl_2_) for 1 min, 0.5% Triton X-100 in CSK buffer for 1 min and 4% PFA for 10 min at room temperature; Cyclin A and CldU: 4% PFA for 10 min and 0.3% Triton Χ-100 in PBS for 5 min at room temperature; S9.6: methanol for 10 min on ice and 0.5% Triton X-100 in PBS for 5 min at room temperature. RNase H (New England Biolabs) was used at 0.05 U μl^−1^ for 36 h at 37 °C. RNase A (Invitrogen) was used at 0.05 ng μl^−1^–2 μg μl^−1^ for 2 h at 37 °C. Samples were blocked with 3% BSA/10% fetal bovine serum. Primary antibodies were mouse anti-phospho-Histone H2AX (Ser139) (JBW301, Millipore 05-636, 1:1,000), rabbit anti-53BP1 (Bethyl A300-272A, 1:30,000), mouse anti-Cyclin A (6E6, Thermo Scientific MS1061, 1:50), rat anti-CldU (BU1/75, AbD Serotec OBT0030G, 1:250) and mouse anti-RNA/DNA hybrid (S9.6, gift from Professor Richard Gibbons, hybridoma supernatant 1:100). Secondary antibodies were anti-mouse IgG AlexaFluor 488 and anti-rabbit IgG AlexaFluor 555 (Molecular Probes). DNA was counterstained with 4,6-diamidino-2-phenylindole (DAPI) and images acquired as above. For quantification of nuclear S9.6 intensity, ImageJ was used to generate nuclear masks based on DAPI staining and mean S9.6 fluorescence intensities per pixel were quantified per nucleus.

### EU incorporation assay

EU incorporation assays were performed using the Click-iT RNA Alexa Fluor 594 Imaging Kit (Invitrogen) according to the manufacturer's instructions. Cells were incubated with 1 mM EU for 1 h, fixed with 4% PFA for 15 min at room temperature, permeabilized with 0.5% Triton X-100 for 15 min and Click-iT reaction was performed. DNA was counterstained with DAPI and images were acquired as above. ImageJ was used to generate nuclear masks based on DAPI staining and mean AlexaFluor 594 fluorescence intensities per pixel were quantified per nucleus.

### siRNA and DNA transfection

siRNAs against TBP (TBPsi #1: sense 5′-GAAUCUUGGUUGUAAACUU-3′ and TBPsi #2: sense 5′-GGAUAAGAGAGCCACGAAC-3′) were purchased from Ambion and Dharmacon. siRNA against RNaseH1 (s48357) was purchased from Ambion. ‘Allstars negative control siRNA' was purchased from Qiagen. Cells were transfected with 50 nM siRNA using Dharmafect 1 reagent (GE Dharmacon). For RNaseH1 overexpression, cells were transfected with 2.5 μg pCMV6-AC-RNase H1-GFP (Origene) using TransIT-2020 (Mirus Bio). Empty pcDNA 3.1 (+) vector was purchased from Invitrogen.

### Western blotting

Cell extracts were prepared in UTB buffer (50 mM Tris-HCl pH 7.5, 150 mM β-mercaptoethanol and 8 M urea) and sonicated to release DNA-bound proteins. Primary antibodies used were mouse anti-HRAS (Santa Cruz sc-29, 1:500), mouse anti-TBP (1TBP18, Abcam ab818, 1:2,000), rabbit anti-RNaseH1 (Abcam ab83179, 1:3,000), rabbit anti-phospho-ERK1/2 (Cell Signaling 9101, 1:1,000), rabbit anti-ERK1/2 (Cell Signaling 9102, 1:1,000), rabbit anti-γH2AX (Bethyl A300-081A, 1:1,000), rabbit anti-phospho-S345 CHK1 (Cell Signaling 2341, 1:1,000), rabbit anti-CHK1 (Cell Signaling 2345S, 1:1,000), rabbit anti-phospho-S33 RPA32 (Bethyl A300-426A, 1:1,000), mouse anti-RPA32 (9H8, Abcam ab2175, 1:2,000), rabbit anti-Turbo-GFP (Evrogen AB513, 1:10,000), mouse anti-p53 (DO-1, gift from Professor David Lane, 1:100), mouse anti-CyclinB1 (V152, Abcam ab72, 1:1,000), mouse anti-αTUBULIN (B512, Sigma T6074, 1:10,000), rabbit anti-βACTIN (Cell Signaling A967S, 1:5,000) and mouse anti-GAPDH (6C5, Abcam ab8245, 1:10,000).

### ChIP analysis

ChIP was performed using 5 μg of anti-γH2AX (Merck Millipore, 07-164) and H2AX (Merck Millipore, 07-627) antibodies as previously described^62^,^63^. Where indicated, samples were treated with 0.5 μM Aphidicolin (Sigma) for 2 h before ChIP. The immunoprecipitated (γH2AX or H2AX antibody in immunoprecipitation (IP) reaction), control (beads only) and input DNAs were used as templates for qPCR, containing QuantiTect SYBR Green PCR Master Mix (QIAGEN) and gene-specific primers (see Supplementary Table 1).

### RNA/DNA hybrid immunoprecipitation

DIP analysis was performed with RNA/DNA hybrid antibody (0.3 μg μl^−1^ per IP reaction) purified from S9.6 hybridoma cell lines as previously described^33^. The immunoprecipitated (S9.6 antibody in IP reaction), control (beads only) and input DNAs were used as templates for qPCR. DIP RNase H-sensitivity analysis was carried out before IP step with the addition of 25 U RNase H (NEB, M0297S). One hundred microlitres of nuclease digestion reaction contained 1 × reaction buffer and it was performed for 3 h at 37 °C.

### Slot-blot experiments

Slot-blot experiments were carried out as described^32^. Genomic DNA (1.2 μg) were treated with 2 U of RNase H per μg of DNA (NEB, M0297S) for 2 h at 37 °C before loading on the slot blot. Half of the DNA sample was probed with S9.6 antibody (1:1,000) and the other half with anti-ssDNA antibody (MAB3031, Millipore, 1:25,000) as described^32^. Secondary antibody was goat anti-mouse horseradish peroxidase (1:10,000). Images were acquired with LAS-4000 (Fujifilm) and quantified using Image Studio Lite software (Li-COR Biosciences).

### Cell proliferation and β-galactosidase assays

For proliferation assays, 1 × 10^5^ or 2 × 10^5^ cells were seeded in 12-well plates and incubated with 10 mg ml^−1^ resazurin for 2 h at the indicated time points. Resorufin fluorescence at 590 nm was measured using a BMG Labtech PHERAstar FS microplate reader. Cell numbers were determined by multiplying the number of initially seeded cells with (FR_n_/FR_0_) (FR_n_: fluorescence reading at time point, FR_0_: initial fluorescence reading). β-Galactosidase staining was performed using the Senescence β-Galactosidase Staining Kit (Cell Signaling) according to the manufacturer's instructions.

### Nucleotide quantification

Cells were harvested and nucleotides extracted with 70% ice-cold methanol. Precipitated proteins were removed by centrifugation and supernatants stored at −80 °C. Supernatants were dried in a heated vacuum centrifuge and reconstituted in HPLC starting eluent. Samples were analysed by HPLC (Waters 2695, Watford, UK) with a photodiode array detector (Waters 2996). Separation was achieved using an Ace C18 (3 μm, 3 × 125 mm, Hichrom, UK) column maintained at 35 °C with eluent A: 10 mM potassium dihydrogen phosphate and 10 mM tetrabutylammonium hydrogen sulfate, 10% methanol pH 6.9; eluent B: 50 mM potassium dihydrogen phosphate, 6 mM tetrabutylammonium hydrogen sulfate and 30% methanol pH 7, using a flow rate of 0.6 ml min^−1^ and a gradient of 25–80% B over 25 min, with a run time of 30 min. Nucleotides were identified by comparing with absorbance spectra and retention times of commercially available standards.

### Quantitative real-time PCR

Total RNA was harvested using TRIZOL reagent (Invitrogen) followed by DNase I treatment (Roche), 1.5 μg of total RNA was reverse transcribed using SuperScript Reverse Transcriptase III (Invitrogen) with random hexamers (Invitrogen), following manufacturer's instructions. The qPCR primers for amplification are listed in Supplementary Table 1. For quantitative real-time PCR, 2 μl of cDNA was analysed using a Rotor-Gene RG-3000 real-time PCR machine (Corbett Research) with QuantiTect SYBR green (Qiagen). Cycling parameters were 95 °C for 15 min, followed by 45 cycles of 94 °C for 20 s, 58 °C–62 °C for 20 s and 72 °C for 20 s. Fluorescence intensities were plotted against the number of cycles by using an algorithm provided by the manufacturer.

### Pulsed field gel electrophoresis

Cells (2 × 10^6^) per sample were treated as indicated, harvested and melted into 1.0% InCert-Agarose (Lonza) inserts. Inserts were digested in 0.5 M EDTA-1% *N*-laurylsarcosyl-proteinase K (1 mg ml^−1^) at room temperature for 48 h and washed three times in TE buffer. Inserts were loaded onto a separation gel (1% chromosomal-grade agarose, Bio-Rad). Separation was performed using a CHEF DR III (BioRad; 120 field angle, 240 s switch time, 4 V cm^−1^, 14 °C) for 20 h. Images of ethidium bromide-stained gels were acquired using a Syngene G:BOX gel imaging system. DSBs (chromosome fragments >2 Mb) were quantified by densitometry using ImageJ. Intensity of DNA entering the gel was normalized to total DNA and untreated control to obtain final values.

### Statistical analysis

Unless stated otherwise, all values are means ±1 s.e.m. of results from independent biological repeats. Scatter plots show pooled data, but numerical values displayed on plots represent the means ±1 s.e.m. of the results from independent repeats. Numbers of repeats *N* are indicated in the figure legends. Statistical tests were performed using the one-tailed Student's *t*-test. Coexpression data were obtained using CBioPortal^61^,^64^ and statistical analyses were performed using the Student's *t*-test based on *t*=(r*√*n*−2)/(√−*r*^2^) where *r* is Pearson's coefficient and *n* is number of samples in analysis.

### Data availability

The authors declare that all the data supporting the findings of this study are available within the article and its Supplementary Information files and from the corresponding authors upon reasonable request.

## Additional information

**How to cite this article:** Kotsantis, P. *et al*. Increased global transcription activity as a mechanism of replication stress in cancer. *Nat. Commun.* **7,** 13087 doi: 10.1038/ncomms13087 (2016).

## Supplementary Material

Supplementary Information Supplementary Figures 1-10, Supplementary Table 1

## Figures and Tables

**Table 1:** TCGA data sets showing correlation between mRNA expression of *TBP* or *RNASEH1* with oncogenes and replication stress markers.

**TCGA** **data set**	***MYC***	***RAS***	***CHEK1***	***CHEK2***
*TBP mRNA expression correlation with:*				
Colorectal adenocarcinoma		0.41 (5%)**	0.54 (1%)**	0.42 (2%)**
Glioblastoma multiforme	0.41 (5%)**	0.33 (10%)*	0.47 (1%)**	
Stomach adenocarcinoma		0.34 (10%)**	0.42 (5%)**	0.45 (1%)**
Lung adenocarcinoma		0.39 (1%)**	0.37 (1%)**	
Lung squamous cell carcinoma				0.32 (5%)*
Bladder urothelial carcinoma	0.46 (1%)**	0.34 (5%)*		
Acute myeloid leukemia	0.31 (5%)*			0.30 (10%)*
Skin cutaneous melanoma		0.31 (1%)**		
Pheochromocytoma and Paraganglioma	0.31 (5%)*	−0.36 (5%)*		
Liver hepatocellular carcinoma			0.45 (1%)**	
*RNASEH1 mRNA expression correlation with:*				
Colon adenocarcinoma			0.48 (1%)**	
Glioblastoma multiforme		0.71 (1%)**		
Lung adenocarcinoma		0.5 (1%)**	0.48 (1%)**	0.38 (5%)**
Lung squamous cell carcinoma		0.31 (5%)*	0.44 (1%)**	0.36 (1%)*
Breast invasive carcinoma		0.37 (10%)**	0.57 (1%)**	0.4 (5%)**
Pheochromocytoma and Paraganglioma		0.38 (10%)**		
Prostate adenocarcinoma		0.3 (10%)**		
Pancreatic adenocarcinoma			0.48 (1%)**	
Thyroid carcinoma		0.51 (5%)**		
Brain lower grade glioma		−0.37 (10%)**		

Blank cells, no correlation; *MYC*, *CMYC/NMYC*; *RAS*, *NRAS/HRAS/KRAS*; TBP, TATA-box binding protein.

Values: Pearson's coefficient *r* (0.3–0.5: positive correlation, 0.5–1: strong positive correlation, −0.3–−0.5: negative correlation, empty cell: no correlation). Values in brackets: top % of all genes; *P<0.00005 and **P<0.000001, Student's *t*-test.
