# Supplementary material for: Increased global transcription activity as a mechanism of replication stress in cancer
Source: Nat Commun. 2016 Oct 11;7:13087. doi: 10.1038/ncomms13087 (PMC5062618; doi:10.1038/ncomms13087)
Supplement: Supplementary Information — Supplementary Figures 1-10, Supplementary Table 1 [file ncomms13087-s1.pdf]

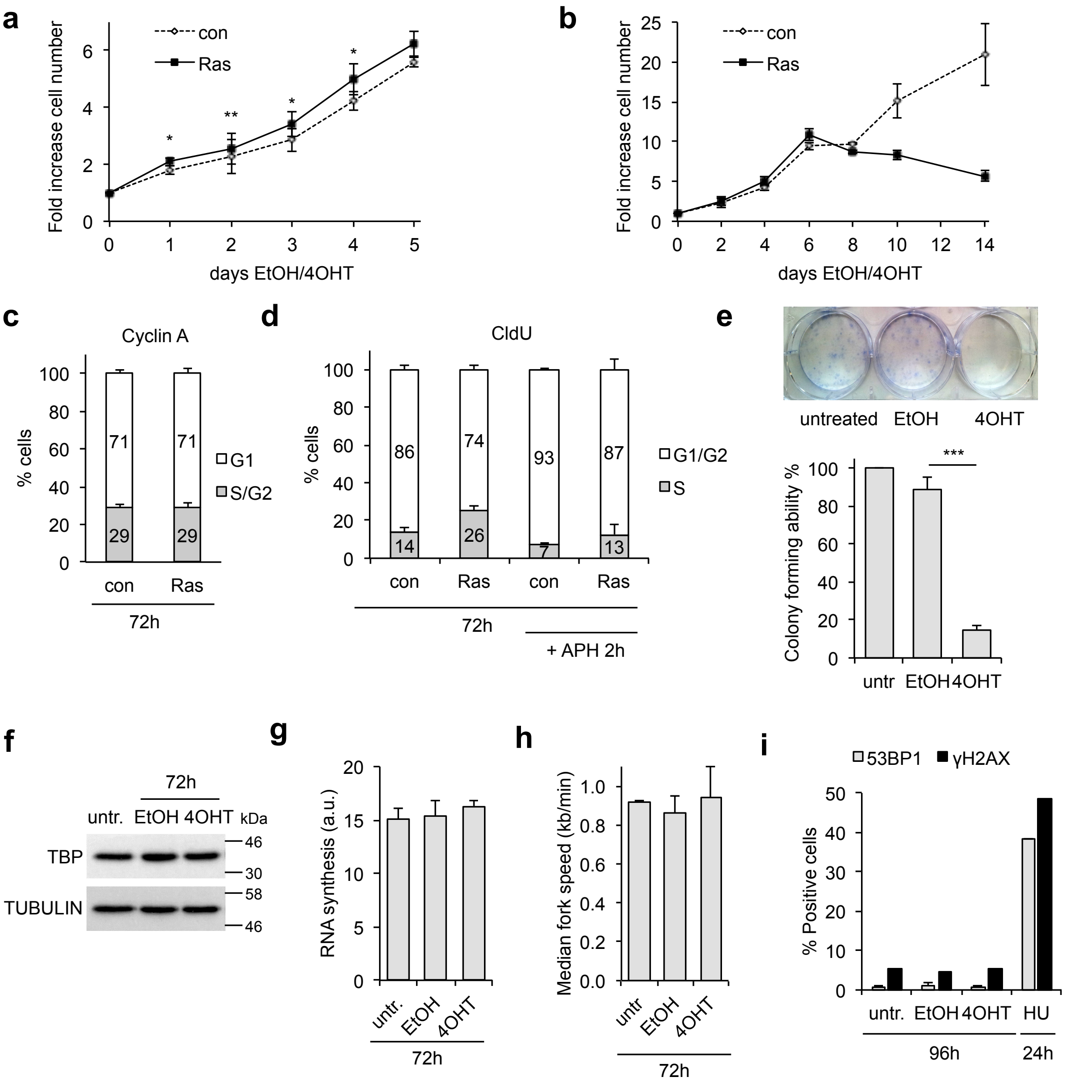

Supplementary Figure 1. Altered proliferation and replication stress in BJ-HRASV^{12} cells.

(**a**), (**b**) Proliferation of BJ-HRASV^{12} cells treated with EtOH or 4OHT measured by resazurin reduction assay. N = 4 (a), N = 2 (b). (**c**) S/G2 phase percentages after RAS induction for 72 h as determined by Cyclin A staining. N = 4. (**d**) S phase percentages after RAS induction for 72 h as determined by labelling with 25 µM CldU for 20 min. + APH: Treatment with 0.5 µM Aphidicolin for 2 h prior to and during CldU labelling. N = 3. (**e**) Colony formation of BJ-HRASV^{12} cells after incubation with no treatment, EtOH or 4OHT for 13 days. Above: Representative images of colonies. N = 4. (**f**) Protein levels of TBP and TUBULIN (loading control) in BJ-hTert cells treated with EtOH, 4OHT or untreated for 72 h. (**g**) Nascent RNA synthesis measured by EU incorporation in BJ-hTert cells treated with EtOH, 4OHT or untreated for 72 h. N = 2. (**h**) Average replication fork speeds in BJ-Tert cells treated with EtOH, 4OHT or untreated for 72 h. N = 2. (**i**) Percentages of BJ-hTert cells with more than 8 53BP1 (N = 2) or γH2AX (N = 1) foci after treatment with EtOH, 4OHT or untreated for 96 h. 2 mM hydroxyurea (HU) for 24 h was used as a positive control (N = 1). Means +/-SEM (bars) are shown. Student's t-test, *p<0.05, **p<0.01, ***p<0.001.

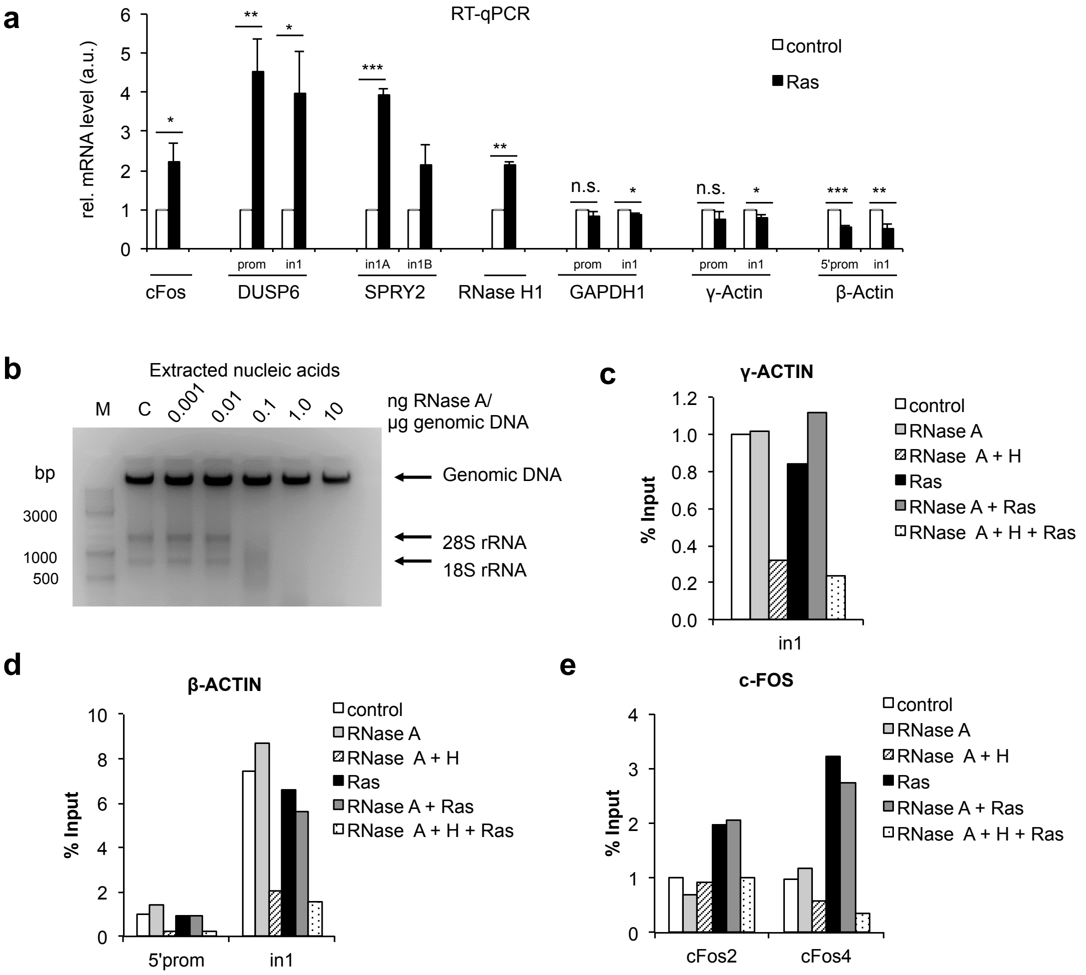

Supplementary Figure 2. Gene expression analysis and RNase A pre-treatment for R-loop DIP in HRAS^{V12} cells.

(**a**) RNA levels of cFos, DUSP6, SPRY2, RNase H1, GAPDH, γ-Actin and β-Actin genes in HRAS^{V12} cells, as determined by qRT-PCR. Values are relative to control cells for each amplicon. N = 4. (**b**) Agarose gel analysis of RNA trimming by RNase A during DIP. 5 µg of purified genomic DNA was prepared according to the DIP protocol. RNase A enzyme was added to the indicated concentrations and incubated for 2 h at 37°C. Control sample (C) was not treated with RNase A. Samples were treated with proteinase K and extracted nucleic acids were separated on a 1.2% agarose gel. DNA size markers are shown to the left of the gel (lane M). The migration of genomic DNA and ribosomal RNA (18S and 28S rRNA) is indicated on the right of the gel. (**c**) DIP analysis of R-loop induction on the γ-ACTIN, β-ACTIN (**d**) and c-FOS (**e**) genes after RAS induction for 72 h. Genomic DNA samples were pre-treated with 1 ng/µg of RNase A and 1 ng/µg of RNase A + 10 U RNase H for 2 h at 37°C before immunoprecipitation with S9.6 antibody. Values were normalised to the control cells in each amplicon. Means +/- SEM (bars) are shown. Student's t-test, *p<0.05, **p<0.01, ***p<0.001. n.s. is non-significant.

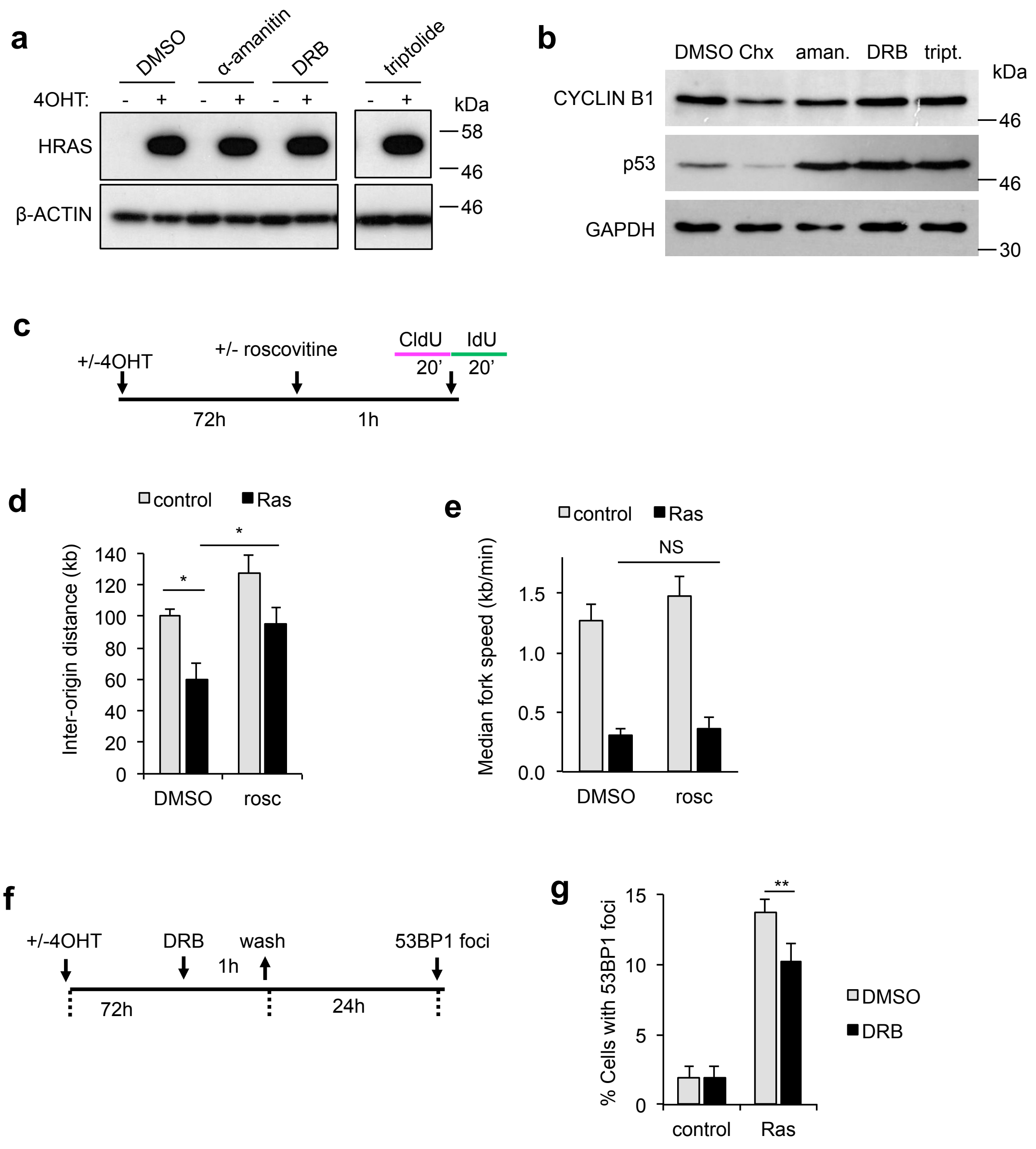

Supplementary Figure 3. Transcription inhibition but not CDK inhibition, rescues replication stress after RAS induction.

(**a**) Protein levels of HRAS and βACTIN in cells treated with DMSO, DRB, or triptolide for 100 min or α-amanitin for 4 h, 72 h after RAS induction. (**b**) Protein levels of CYCLIN B1, p53 and GAPDH (loading control) in BJ-HRASV^{12} cells treated with 100 μg/ml cycloheximide or transcription inhibitors for 100 min. (**c**) 72 h after RAS induction, cells were incubated with 25 μM roscovitine or DMSO for 1 h before and during DNA fibre labelling. (**d**) Average inter-origin distances in cells treated with roscovitine 72 h after RAS induction. N = 3. (**e**) Median fork speeds in cells treated with roscovitine 72 h after RAS induction. N = 3. (**f**) 72 h after RAS induction, cells were incubated with DRB for 100 min, washed, and 53BP1 foci were quantified 24 h later. (**g**) Percentage of cells displaying more than 8 53BP1 foci after DRB treatment. N = 3. Means +/-SEM (bars) are shown. Student's t-test, *p<0.05, **p<0.01.

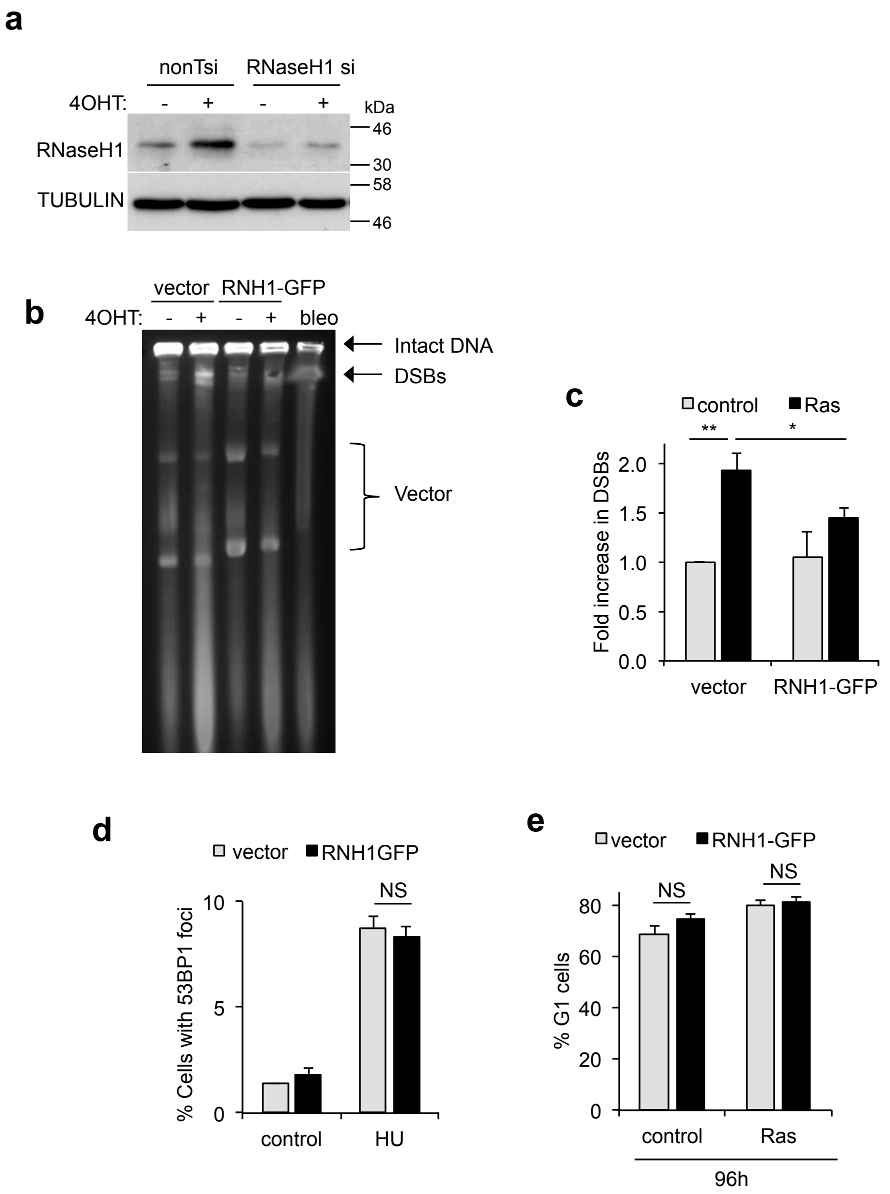

Supplementary Figure 4. RNaseH1 overexpression rescues replication stress after RAS induction.
(**a**) Validation of RNaseH1 antibody. Protein levels of RNaseH1 and TUBULIN (loading control) after 48 h RAS induction followed by transfection with non-targeting (nonTsi) or RNaseH1 siRNA for 24 h. (**b**) Pulse-field gel electrophoresis (PFGE) showing DSB formation +/-RNaseH1-overexpression. 48 h after RAS induction, cells were transfected with pCMV6-AC-RNaseH1-GFP or empty vector, and analysed 48 h later. bleomycin (bleo, 10 µg/ml for 24 h) was used as a positive control. (**c**) Fold increase in DNA released from plugs during PFGE, normalised to control + vector. N = 4. (**d**) Percentages of BJ-HRASV[12] cells containing more than 8 53BP1 foci after transfection with pCMV6-AC-RNaseH1-GFP or empty vector with simultaneous HU (2 mM) treatment for 24 h. N = 2. (**e**) Percentages of BJ-HRASV[12] cells in G1 phase (Cyclin A negative), after transfection with pCMV6-AC-RNaseH1-GFP or empty vector 48 h after RAS induction and Cyclin A staining 48 h later. N = 3. Means +/-SEM (bars) are shown. Student's t-test, *p<0.05, **p<0.01. NS: not significant.

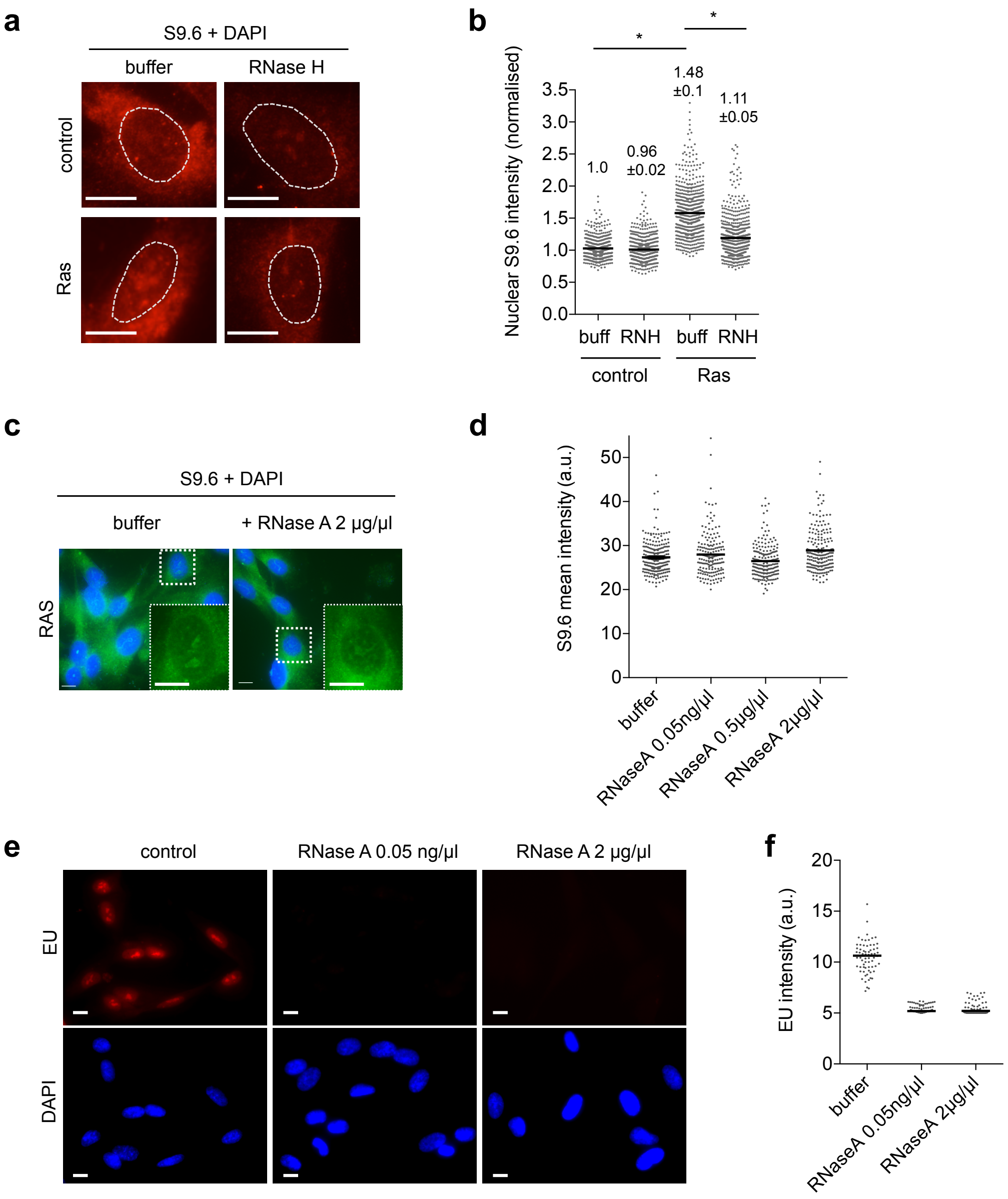

Supplementary Figure 5. Validation of S9.6 quantification by immunofluorescence

(**a**) Representative images of S9.6 immunostaining after RAS induction for 72 h, +/- RNase H treatment. S9.6 staining outside the nucleus is due to mitochondrial R-loops[31, 32]. (**b**) Quantification of nuclear S9.6 intensity after RAS for 72 h, +/- RNase H treatment. N = 3. (**c**) Representative images of S9.6 immunostaining after RAS induction for 72 h, +/- RNase A treatment. (**d**) Percentages of BJ-HRAS^{V12} cells displaying elevated S9.6 immunostaining after RAS induction for 72 h +/- RNase A treatment. N = 2. (**e**) (**f**) Analysis of RNA trimming by RNase A on fixed cells. EU was used to label RNA in live cells for 1 h, cells were fixed and treated with RNase A followed by AlexaFluor 594 Click-IT reaction and fluorescence quantification. N = 1. Means +/- SEM (bars) are shown. Student's t-test, *p<0.05, **p<0.01. Bars: 10 µm.

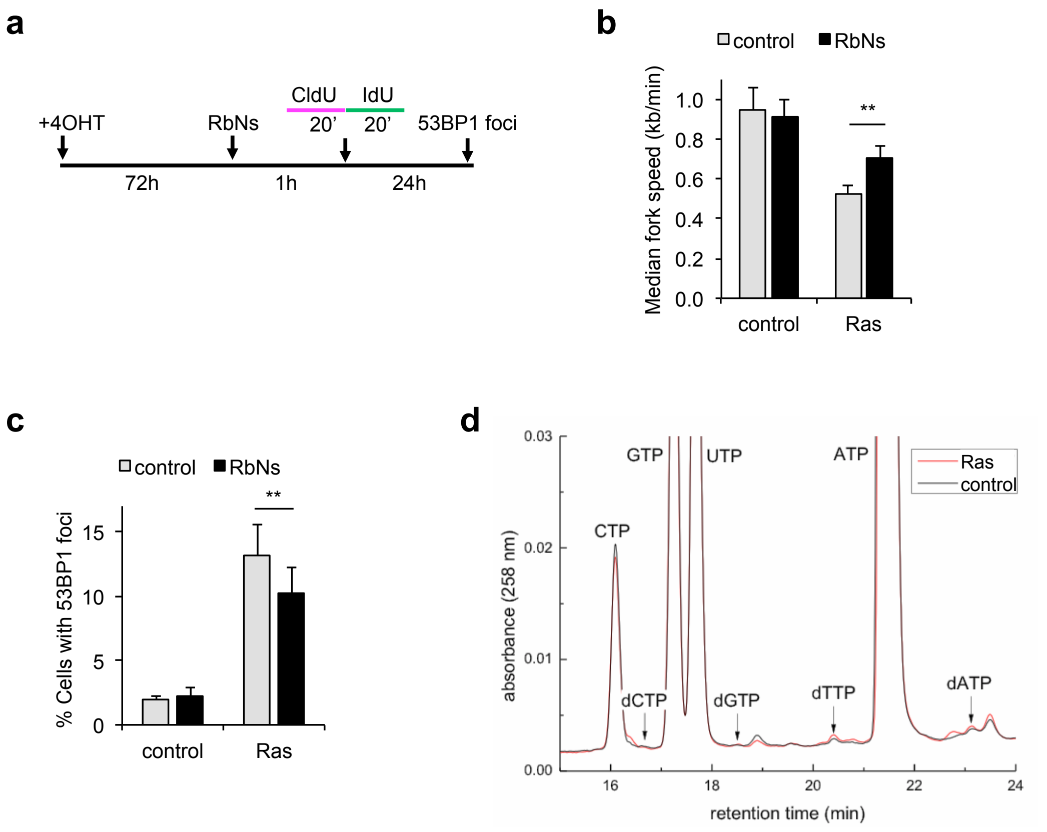

e

	Fold change Ras/control	SEM
CTP	1.10	0.12
GTP	1.09	0.14
UTP	1.00	0.06
ATP	0.87	0.05
dCTP	ND	ND
dGTP	ND	ND
dTTP	~2	ND
dATP	~2	ND

Supplementary Figure 6. Effect of nucleoside supplementation on replication stress after RAS induction.

(**a**) 72 h after RAS induction, BJ-HRASV^{12} cells were treated with ribonucleosides (RbNs) for 100 min prior to and during DNA fibre analysis, or for 24 h prior to 53BP1 foci staining. (**b**) Median replication fork speeds in cells treated with RbNs 72 h after RAS induction. N = 3. (**c**) Percentage of RbNs-treated cells displaying more than 8 53BP1 foci 96 h after RAS induction. N = 3. (**d**) HPLC chromatogram of nucleotides isolated 72 h after RAS induction. (**e**) Fold change in ribonucleotide and deoxyribonuclotide levels 72 h after RAS induction versus control. Means +/-SEM (bars) are shown. Student’s t-test, **p<0.01.

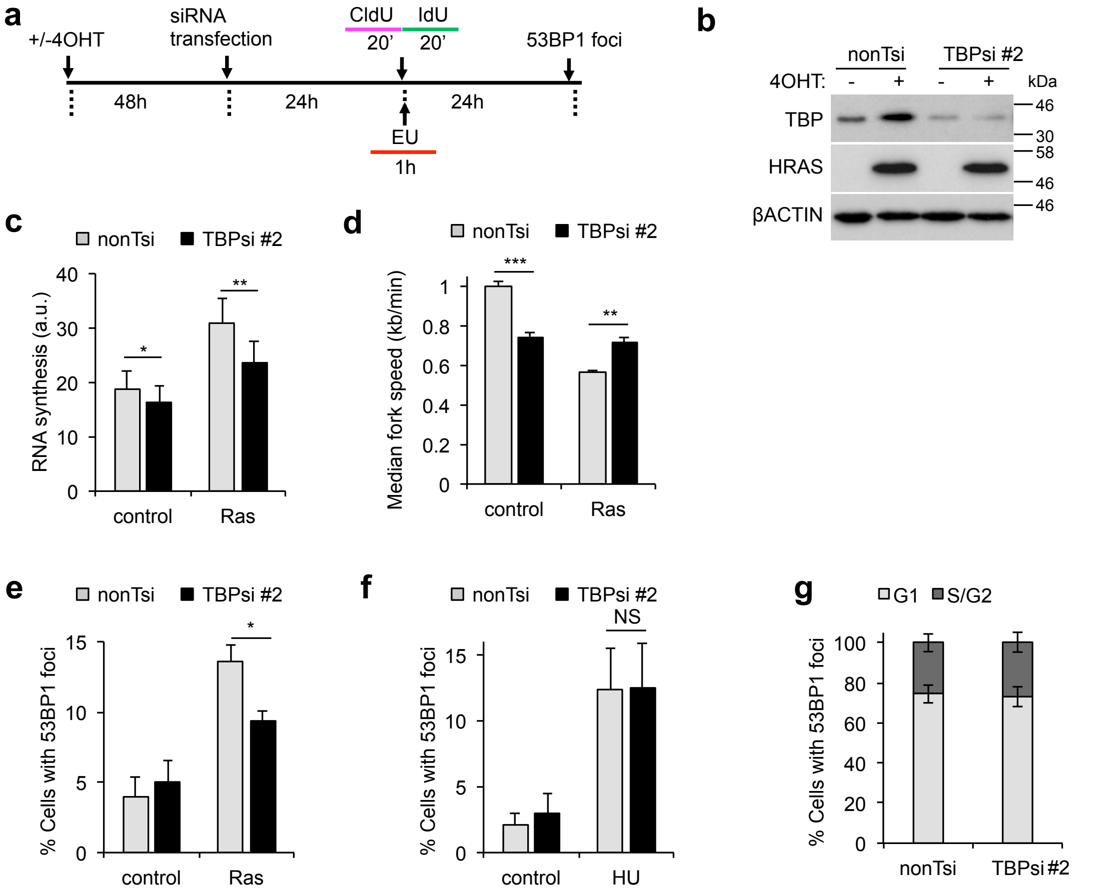

Supplementary Figure 7. TBP promotes replication stress in presence of HRAS^{V12} (TBP siRNA #2).

(**a**) 48 h after RAS induction, BJ-HRASV^{12} cells were transfected with TBP siRNA (TBPsi #2) or control siRNA (nonTsi) and processed for EU, DNA fibre analysis or Western blot 24 h later and for 53BP1 staining 48 h later. (**b**) Protein levels of TBP, HRAS and βACTIN (loading control) 72 h after RAS induction and 24 h after siRNA transfection. (**c**) Quantification of nascent RNA synthesis by EU incorporation +/- TBPsi #2 72 h after RAS induction. N = 4. (**d**) Median replication fork speeds +/- TBPsi #2 72 h after RAS induction. N = 4. (**e**) Percentages of cells containing more than 8 53BP1 foci, +/- TBPsi #2 96 h after RAS induction. N = 3. (**f**) Percentages of cells +/- TBPsi #2 containing more than 8 53BP1 foci after treatment with 2 mM HU for 24 h. N = 2. (**g**) Cell cycle distribution of 53BP1 foci-positive cells +/- TBPsi #2 determined by co-staining with Cyclin A. N = 2. Means +/-SEM (bars) are shown. Student's t-test, *p<0.05, **p<0.01, ***p<0.001. NS: not significant.

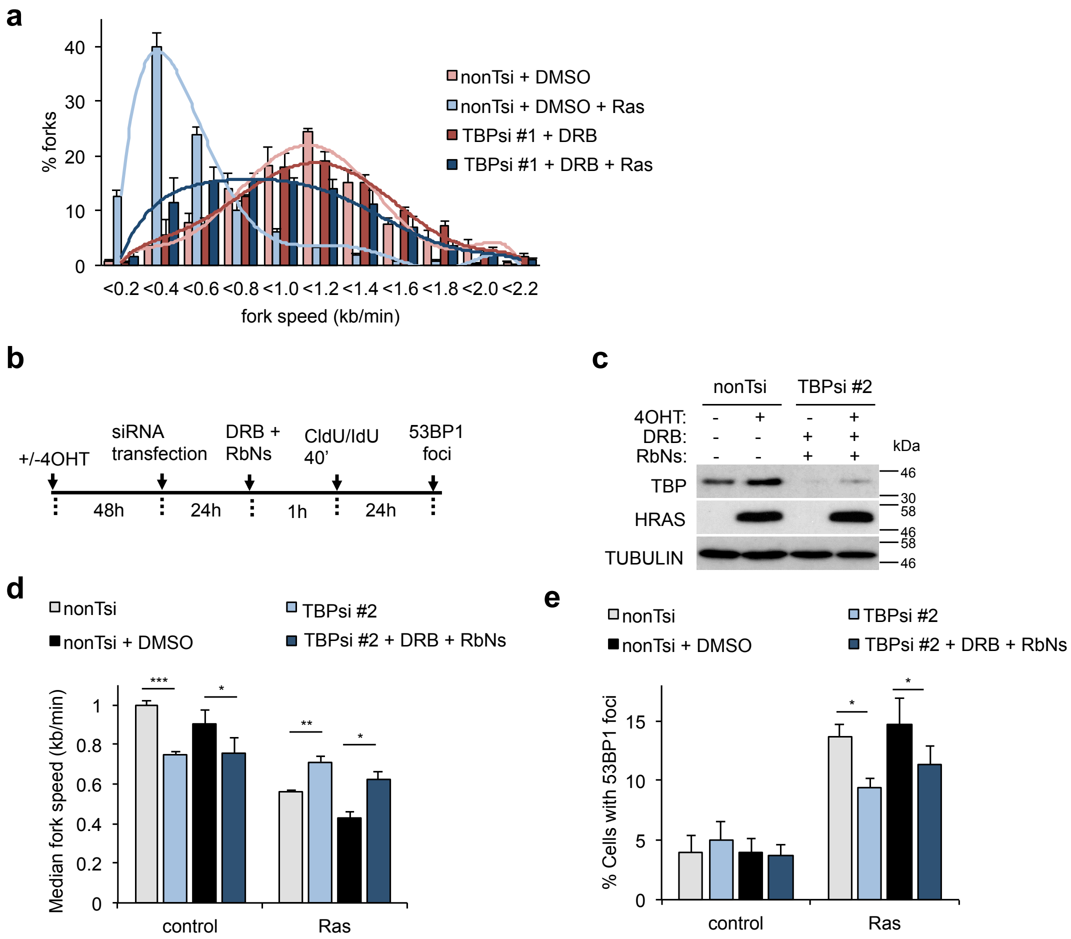

Supplementary Figure 8. Nucleoside supplementation does not rescue replication stress in addition to transcription inhibition.

(**a**) Distributions of fork speeds in cells treated with TBP siRNA #1 and DRB 72 h after RAS induction, as in Fig. 6j. N = 3. (**b**) 48 h after RAS induction, cells were transfected with TBP siRNA #2 (TBPsi #2) or control siRNA (nonTsi). 24 h later, cells were treated with DRB and ribonucleosides (RbNs) for 100 min and processed for DNA fibre analysis and Western blot, or treated with DRB and RbNs for another 24 h prior to 53BP1 staining. (**c**) Protein levels of TBP, HRAS, TUBULIN (loading control) in cells treated with TBP siRNA #2, DRB and RbNs, 72 h after RAS induction. (**d**) Median replication fork speeds in cells treated with TBP siRNA #2, DRB and RbNs 72 h after RAS induction, compared to TBP siRNA #2 alone. N = 3. (**e**) Percentages of cells treated with TBP siRNA #2, DRB and RbNs containing more than 8 53BP1 foci after 96 h after RAS induction, compared to TBP siRNA #2 alone. N = 3. Means +/-SEM (bars) are shown. Student's t-test, *p<0.05, **p<0.01, ***p<0.001.

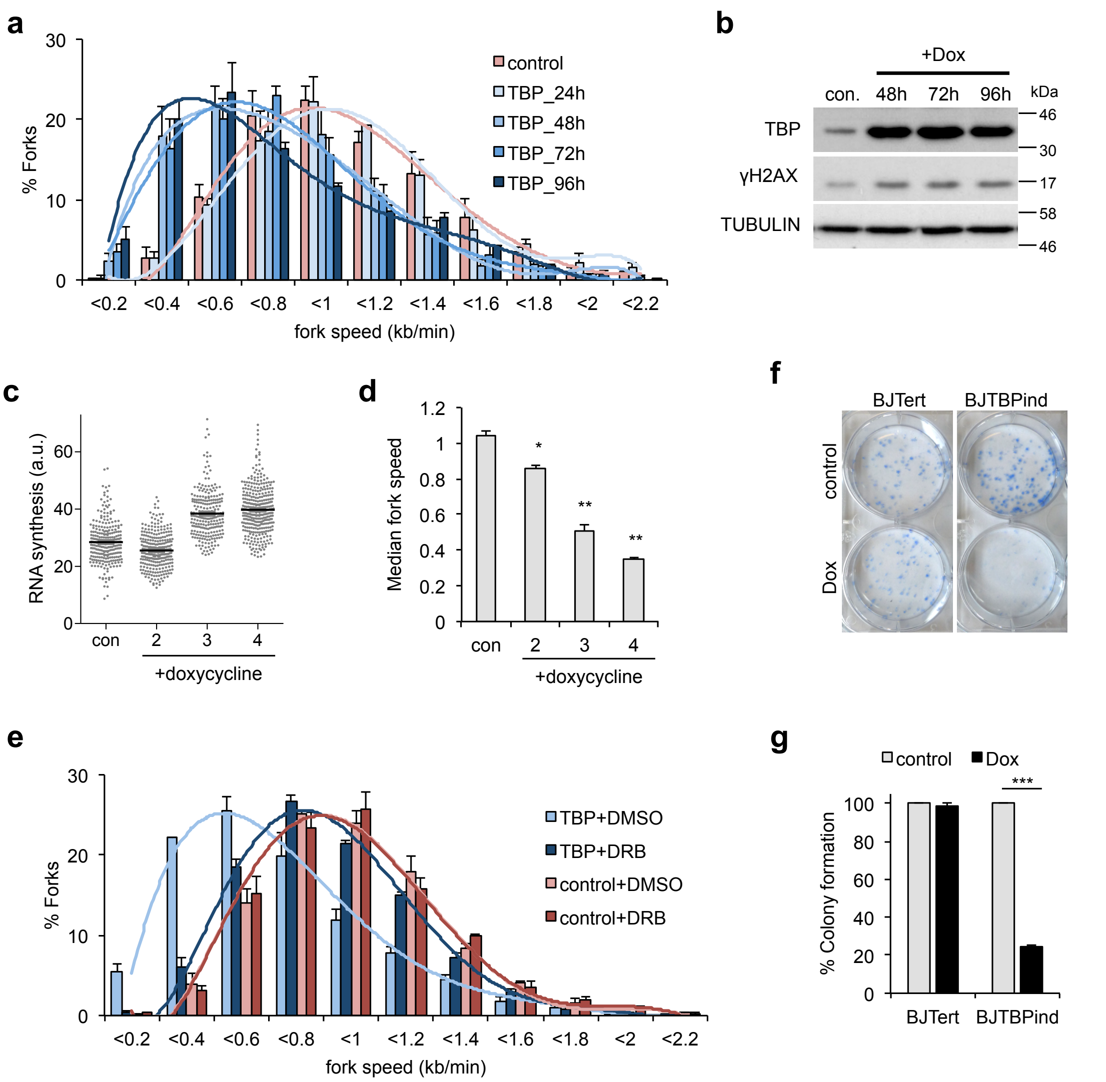

Supplementary Figure 9. TBP overexpression causes replication stress. (**a**) Distributions of fork speeds after TBP overexpression in BJ-TBPind cells as in Fig. 7d. (**b**) Protein levels of TBP, γH2AX and TUBULIN (loading control) in TBP inducible MRC5 (MRC5-TBPind) cells treated with doxycycline for 48 – 96 h. No doxycycline was used as a control. (**c**) Nascent RNA synthesis as measured by EU incorporation in MRC5-TBPind cells after induction of TBP overexpression. N = 1. (**d**) Median replication fork speeds in MRC5-TBPind cells after induction of TBP overexpression. N = 3. (**e**) Distributions of fork speeds in BJ-TBPind cells treated with DRB as in Fig. 7f. N = 3. (**f**) Representative images of colonies and (**g**) quantification of colony formation of BJ-hTert and BJ-TBPind cells after incubation +/- doxycycline for 13 days. N = 2 (BJ-hTert), N = 3 (BJ-TBPInd). Means +/-SEM (bars) are shown. Student's t-test, *p<0.05, **p<0.01, ***p<0.001.

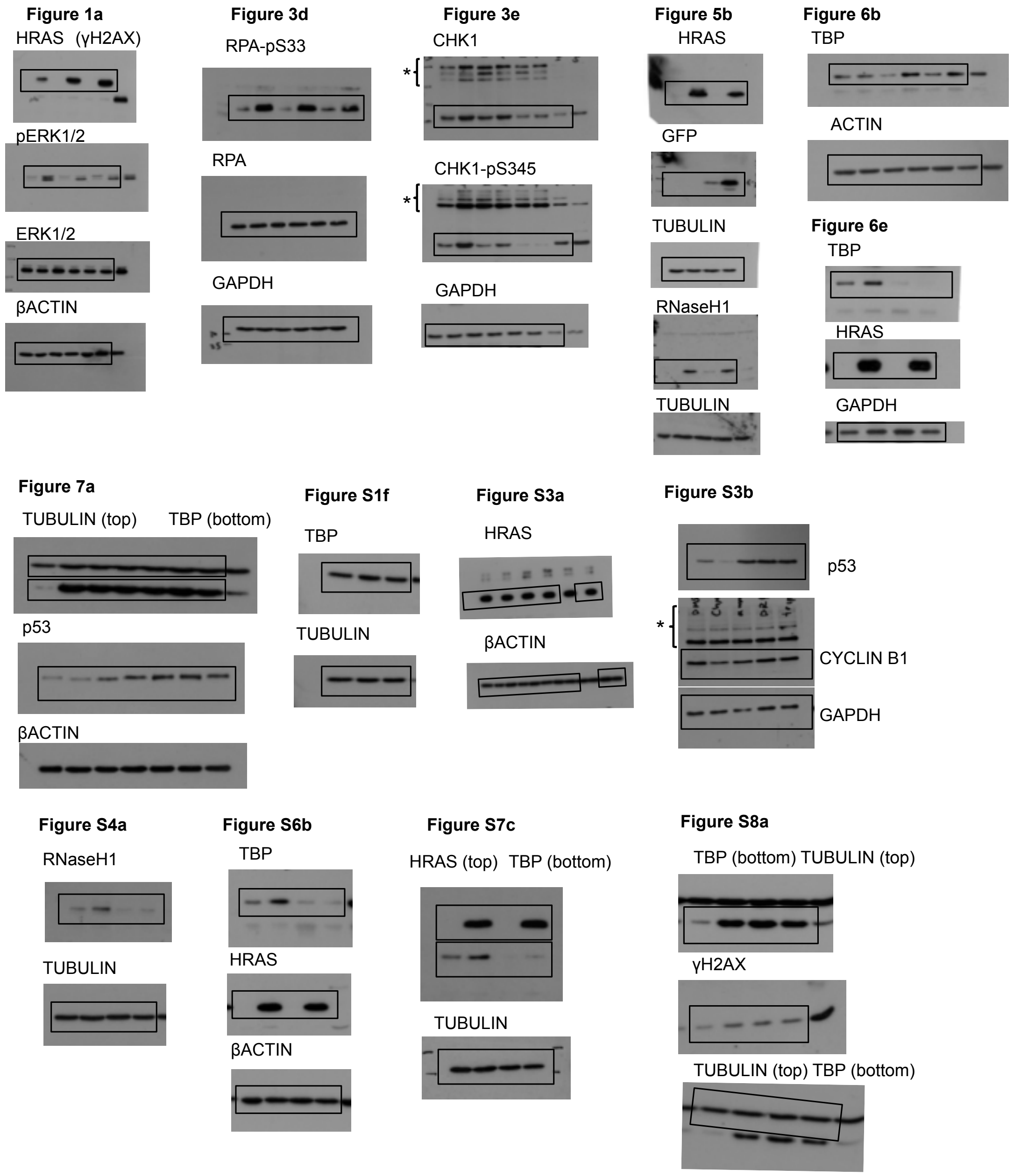

Supplementary Figure 10. Original images of Western blots. Asterisks denote non-specific bands from the indicated antibody. Additional loading controls are included for RNaseH1 (Fig. 5b) and p53 (Fig. 7a) where the samples were re-run on separate gels.

Supplementary Table 1: Sequences of PCR primers

Name	Sequence (5' → 3')
**β-actin gene**	
5'prom (F)	CCA CCT GGG TAC ACA CAG TCT
5'prom (R)	TGT CCT TGT CAC CCT TTC TTG
prom (F)	GAG GGG AGA GGG GGT AAA
prom (R)	AGC CAT AAA AGG CAA CTT TCG
in1 (F)	CGG GGT CTT TGT CTG AGC
in1 (R)	CAG TTA GCG CCC AAA GGA C
5'pause (F)	TTA CCC AGA GTG CAG GTG TG
5'pause (R)	CCC CAA TAA GCA GGA ACA GA
pause (F)	GGG ACT ATT TGG GGG TGT CT
pause (R)	TCC CAT AGG TGA AGG CAA AG
**c-FOS gene**	
c-FOS2 (F)	TTC CAC GCT TTG CAC TGAA
c-FOS2 (R)	GGA CTT AAG CCT CGG CTC
c-FOS3 (F)	TGA GCC CGT GAC GTT TAC
c-FOS3 (R)	TGC AGA TGC GGT TGG AG
c-FOS4 (F)	GGA TAG CCT CTC TTA CTA CCA CTC A
c-FOS4 (R)	GGA AGC CAG CCT TAC CT
c-FOS nascent (F)	CCA TGA CAG GAG GCC GA
c-FOS nascent (R)	ACA TTC CCA GGA AGA GTA
**Intergenic region**	
Intergenic region (F)	TCC TCC ACC CTC CAA GT
Intergenic region (R)	TGA GGG CTG GTT CGG TC
**DUSP6 gene**	
DUSP6 prom (F)	GGC TGA GTC CAA GAG ATA GC
DUSP6 prom (R)	GCT TCT AAT CCC TCC CTC CA
DUSP6 in1 (F)	AAC GGG TTC TGC GCC TTC TT
DUSP6 in1 (R)	TGT GCC AAT TTG CAT CCC CAA
**SPRY2 gene**	
SPRY2 prom (F)	TTC CCA TTC GCT CAT CTG CCA
SPRY2 prom (R)	AAG CGC ACG CGG AGT ATT TC
SPRY2 in1A (F)	CGA ATT CGG CGC TGA GAG
SPRY2 in1A (R)	ACA GGT TAG AAA TGC GGG
SPRY2 in1B (F)	TCT GTG TCC GTT TGC CCC A
SPRY2 in1B (R)	TGA AAG TGC TTT GAA ACC CCC
**GAPDH gene**	
GAPDH prom (F)	AGC TCA GGC CTC AAG ACC TTG GGC T
GAPDH prom (R)	GGC TGA CTG TCG AAC AGG AGC
GAPDH in1 (F)	CCA CTA GGC GCT CAC TGT TC
GAPDH in1 (R)	TCG TAG ACG CGG TTC GG

**γ-Actin gene**	
γ-Actin prom (F)	GGA AAG ATC GCC ATA TAT GGA C
γ-Actin prom (R)	TCA CCG GCA GAG AAA CGC GAC
γ-Actin in1 (F)	CCG CAG TGC AGA CTT CCG AG
γ-Actin in1 (R)	CGG GCG CGT CTG TAA CAC GG
**RNase H1 gene**	
RNase H1 (F)	TAA CTG GGT TCA AGG TTG GAA G
RNase H1 (R)	TCT TCC GAT TGT TTA GCT CCT TC
**TBP gene**	
TBP (F)	TTC GGA GAG TTC TGG GAT TG
TBP (R)	CTC ATG ATT ACC GCA GCA AA
